# PI(4,5)P2 alleviates colitis by inhibiting intestinal epithelial cell pyroptosis through NNMT-mediated RBP4 m6A modification

**DOI:** 10.1038/s41419-024-07276-3

**Published:** 2024-12-20

**Authors:** Qingfan Yang, Na Diao, Fei Ma, Zicheng Huang, Minzhi Lin, Xinyu Liu, Qin Guo, Pan Li, Jian Tang, Xiang Gao, Kang Chao

**Affiliations:** 1 Department of Gastroenterology, The Sixth Affiliated Hospital, Sun Yat-sen University, Guangzhou, China; 2 Biomedical Innovation Center, The Sixth Affiliated Hospital, Sun Yat-sen University, Guangzhou, China; 3 Maternal & Child Health Research Institute, Zhuhai Center for Maternal and Child Health Care, Zhuhai, China; 4 Institute of Clinical Pharmacology, School of Pharmaceutical Sciences, Sun Yat-Sen University, Guangzhou, China; 5 School of Medicine, Jianghan University, Wuhan, China

**Keywords:** Crohn's disease, Cell death

## Abstract

Lipid metabolism disorder is a critical feature of Crohn’s disease (CD). Phosphatidylinositol (PI) and its derivative, phosphatidylinositol bisphosphate (PIP2), are associated with CD. The mechanisms underlying such association remain unknown. In this study, we explored the role played by the major PI derivative, phosphatidylinositol 4,5-bisphosphate [PI(4,5)P2], in CD pathogenesis. The relationship between CD activity and PI or PIP2 was analyzed via lipidomics. The mucosal expression of PI(4,5)P2 in patients with CD was measured using immunofluorescence. The function and mechanism of PI(4,5)P2 were examined in dextran sulfate sodium (DSS)-induced colitis mice and lipopolysaccharide (LPS)-induced Caco-2 cell models, along with MeRIP and mRNA sequencing. The results suggested lipid PI and PIP2 were substantially negatively associated with disease activity and high-sensitivity C-reactive protein. PI(4,5)P2 was substantially downregulated in the inflamed mucosa of patients with CD. PI(4,5)P2 alleviated mouse colitis, with improvements in survival rate, colon length, weight, and disease activity index. PI(4,5)P2 also alleviated DSS-induced tissue damage, tight junction loss, and intestinal epithelial cell (IEC) pyroptosis. In the in vitro LPS-induced cell model, PI(4,5)P2 inhibited pyroptosis, as well as NLRP3, and caspase-1 expression, in addition to reducing interleukin (IL)-18, IL-1β, and lactate dehydrogenase (LDH) secretion. PI(4,5)P2 mediated NNMT upregulation in mice and Caco-2 cells and suppressed pyroptosis in IECs. NNMT knockdown restricted the inhibitory effect of PI(4,5)P2 on IEC pyroptosis. NNMT inhibited the stability of *RBP4* mRNA via m6A modification, thereby preventing pyroptosis following PI(4,5)P2 treatment. Significant correlations were also observed between PI(4,5)P2 and NNMT, NNMT and RBP4, and RBP4 and GSDMD expression in the intestinal tissues from patients with CD. Our results indicated that PI(4,5)P2 ameliorates colitis by inhibiting IEC pyroptosis via NNMT-mediated *RBP4* m6A modification. Thus, PI(4,5)P2 shows potential as a therapeutic target in CD.

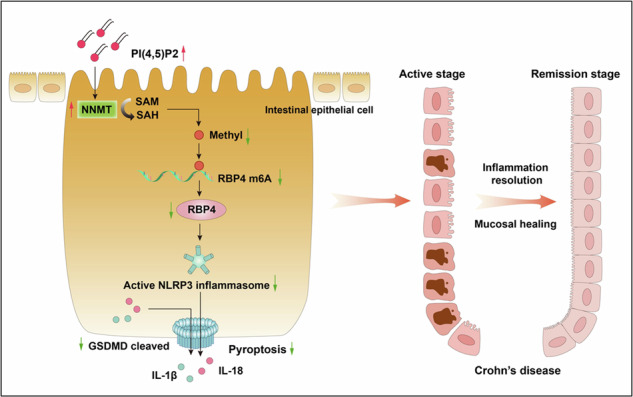

## Introduction

Crohn’s disease (CD) is a progressive inflammatory condition regarded as incurable [1]. Consequently, therapeutic efforts focus primarily on achieving clinical remission and mucosal healing. Although strategies, including biologics, may delay disease progression, the mucosal healing rate remains at only 20–40%, with no long-term remission [2]. Thus, novel therapeutic methods aimed at CD are urgently needed.

Sustained lipid metabolic homeostasis is necessary for maintaining intestinal health. Disordered lipid metabolism is an important feature of CD. Metabolomics has shown that recombinant fatty acid desaturase 2 (FADS2) induced fatty acid desaturation disturbances and lipid mediator imbalance in mesenteric adipocytes are responsible for the chronic inflammation observed in CD [3]. Prior research highlights the involvement of bile acid biosynthesis, arachidonic acid metabolism, and sphingolipid pathways in CD progression, with lipid profiles potentially serving as early diagnostic markers [4]. Receptor modulators of lipid sphingosine-1-phosphate have shown promise in randomized clinical trials for CD treatment [5]; however, substantial gaps remain in understanding the lipid-CD relationship [5]. In a previous pilot study, we found that plasma phosphatidylinositol (PI) and its major derivative, phosphatidylinositol bisphosphate (PIP2), levels were substantially elevated in the remission stage compared to active CD [6], suggesting a potential protective function for PI and PIP2. Nevertheless, the molecular mechanisms by which PI and PIP2 influence CD pathology remain largely unexplored.

Phosphatidylinositol 4,5-bisphosphate [PI(4,5)P2], commonly referred to as PIP2, is a major PI derivative that regulates the cytoskeleton, vesicle transport, ion channels, and plasma membrane signal transduction. It plays key roles in cell growth, differentiation, migration, and inflammatory responses [7–9]. PI(4,5)P2 reportedly [10] acts as an epigenetic regulator of rRNA gene transcription. It regulates inflammation by inhibiting NLRP3 inflammasome activation and nuclear factor kappa B (NF-κB) phosphorylation, and suppressing interleukin (IL)-1β, IL-6, and tumour necrosis factor (TNF)-α secretion by macrophages [10]. Ginsenoside Rb1 was found to attenuate ulcerative colitis by modulating the NRF2/PIP2/NLRP3 pathway in mice [11]. These findings suggest that PI(4,5)P2 may possess anti-inflammatory properties relevant to CD pathogenesis, though further evidence is needed. This study aims to investigate the role and mechanisms of PI(4,5)P2 in CD, with the objective of uncovering novel therapeutic targets rooted in lipid metabolism.

## Materials and methods

### Participants and clinical sample collection

This study was approved by the Ethics Committee of the Sixth Affiliated Hospital affiliated with Sun Yat-sen University (approval number 2023ZSLYEC-211). All participants provided informed consent before enrolment in the study. Adult patients with CD between November 2018 and September 2019 were included. The phenotype of CD was classified using the Montreal Classification [12]. Mucosal healing was defined as a simple endoscopic score (SES-CD) ≤ 2 [13]. The CD activity index (CDAI) < 150 [14] was defined as clinical remission.

Blood samples and paraffinized surgical specimens from patients with CD were collected. Detailed inclusion and exclusion criteria, patient demographics, CD phenotypes and sample collection methods, etc., are provided in Supplementary Information.

### PI(4,5)P2 treatment in a dextran sulfate sodium (DSS)-induced colitis model

Ethical approval was provided by the Ethics Committee of our hospital. A 4% DSS solution [15] and a PI(4,5)P2 (No. P4508, Echelon Biosciences, UT, USA) solution were prepared. Male C57BL/6 mice were randomly and blindly divided into five groups (*n* = 5/group): Blank, DSS + normal saline (NS), PI(4,5)P2 high dose enema [DSS + H-PI(4,5)P2, concentration 200 μmol/L], PI(4,5)P2 low dose enema [DSS + L-PI(4,5)P2, concentration 100 μmol/L], and oral [DSS + O-PI(4,5)P2, concentration 100 μmol/L]. From the date of modeling, activity, eating, weight, stool blood, and stool characteristics were observed daily, and the disease activity index (DAI) was scored [16]. Animal experiments were performed four times, and details are provided in Supplementary Information.

### PI(4,5)P2 treatment in lipopolysaccharide (LPS)-induced cell model

To establish the in vitro model, human Caco-2 cells from Procell (CL-0050, China) were treated with 0.5 µg/mL LPS. To explore the effect of PI(4,5)P2 on Caco-2 cells, cells were treated with PI(4,5)P2 at a final concentration of 12.5, 25, 50, 100, and 200 µM, together with LPS. Independent experiments were performed in triplicate.

### Lipidomic analysis

Ultra-high-performance liquid chromatography coupled with tandem mass spectrometry (UHPLC-MS/MS) was used to separate the lipids, and LipidSearch software (Thermo Fisher Scientific, Waltham, MA, USA) was used to identify the lipid species [6].

### Hematoxylin and eosin (H&E) staining

Fixed and sectioned mouse tissues were H&E stained (details in Supplementary Information). The histological score was evaluated by two professional gastrointestinal pathologists. The severity/range of inflammatory infiltration and crypt damage served as score indicators. Each indicator was scored from 0 to 4 [17].

### Transcriptome sequencing

Transcriptome sequencing of two Caco-2 cell groups (control and PI(4,5)P2-treatment) followed by bioinformatics analysis aimed at identifying differentially expressed genes (DEGs) and pathways were performed (Supplementary Information).

### Cell transfection

Caco-2 cells were transfected with siRNA targeted to *NNMT* and *RBP4*, respectively, to evaluate the effects of NNMT and RBP4 (Supplementary Information).

### Methylated RNA immunoprecipitation (MeRIP) sequencing and MeRIP-qPCR

Total RNA was isolated from Caco-2 cells following NNMT overexpression and subjected to MeRIP sequencing and MeRIP-qPCR (Supplementary Information).

### Dual-luciferase reporter assays

The wild-type 3′-untranslated region (UTR) of *RBP4* (RBP4_WT) and mutated 3′-UTR of *RBP4* (RBP4_MUT) sequences were recombined into a dual-luciferase reporter PmirGLOVector (Promega, USA) plasmid. Caco-2 cells were seeded in 24-well plates and, after reaching 60–80% confluency, transfected with dual-luciferase reporters. After transfection for 48 h, both Renilla and firefly luciferase activities were measured using a dual-luciferase reporter assay kit (Promega, USA), according to the manufacturer’s instructions. Independent experiments were performed in triplicate.

### RNA stability assay

The stability or turnover rate of mRNA in cells is usually defined as the time required to degrade 50% of the existing mRNA molecules. To determine *RBP4* mRNA stability, Caco-2 cells were treated with actinomycin D (5 µg/mL) and harvested at the indicated time points. Total RNA was extracted by TRIzol Reagent and analyzed using RT-PCR. The half-life of mRNA was estimated using linear regression analysis. Independent experiments were performed in triplicate.

Immunofluorescence (IF), flow cytometry, immunohistochemistry (IHC), transmission electron microscopy (TEM) observation of the murine colonic ultrastructure, terminal deoxynucleotidyl transferase-mediated dUTP nick end labeling (TUNEL), qRT-PCR, cell counting kit-8 (CCK8), enzyme-linked immunosorbent (ELISA) assay, lactate dehydrogenase (LDH) activity assay, western blot, and fluorescence in situ hybridization (FISH) analyses were also performed. For details, see Supplementary Information.

### Statistical analysis

Statistical analyses were performed using GraphPad Prism 9 (GraphPad Prism, San Diego, CA, USA). Multiple groups were analyzed by one-way analysis of variance, followed by Tukey’s post-hoc test. The data were normally distributed, and variances between groups were similar. Statistical significance was set at *P* < 0 ∙ 05.

## Results

### PI(4,5)P2 is downregulated in patients with CD

The sample size was expanded to validate the observed relationship between PI and PIP2 in CD as indicated by our previous pilot study [6]. The lipidomics analysis included 102 adult patients with CD, with clinical characteristics detailed in Supplementary Table 2 (Supplementary Information). Of these, 19 patients (18.6%) presented with active disease (CDAI > 150), and 62 patients underwent colonoscopy concurrent with plasma collection, with 24 showing mucosal healing (SES-CD ≤ 2). Serological inflammatory and nutrition-related markers were assessed across all participants.

**Fig. 1:**
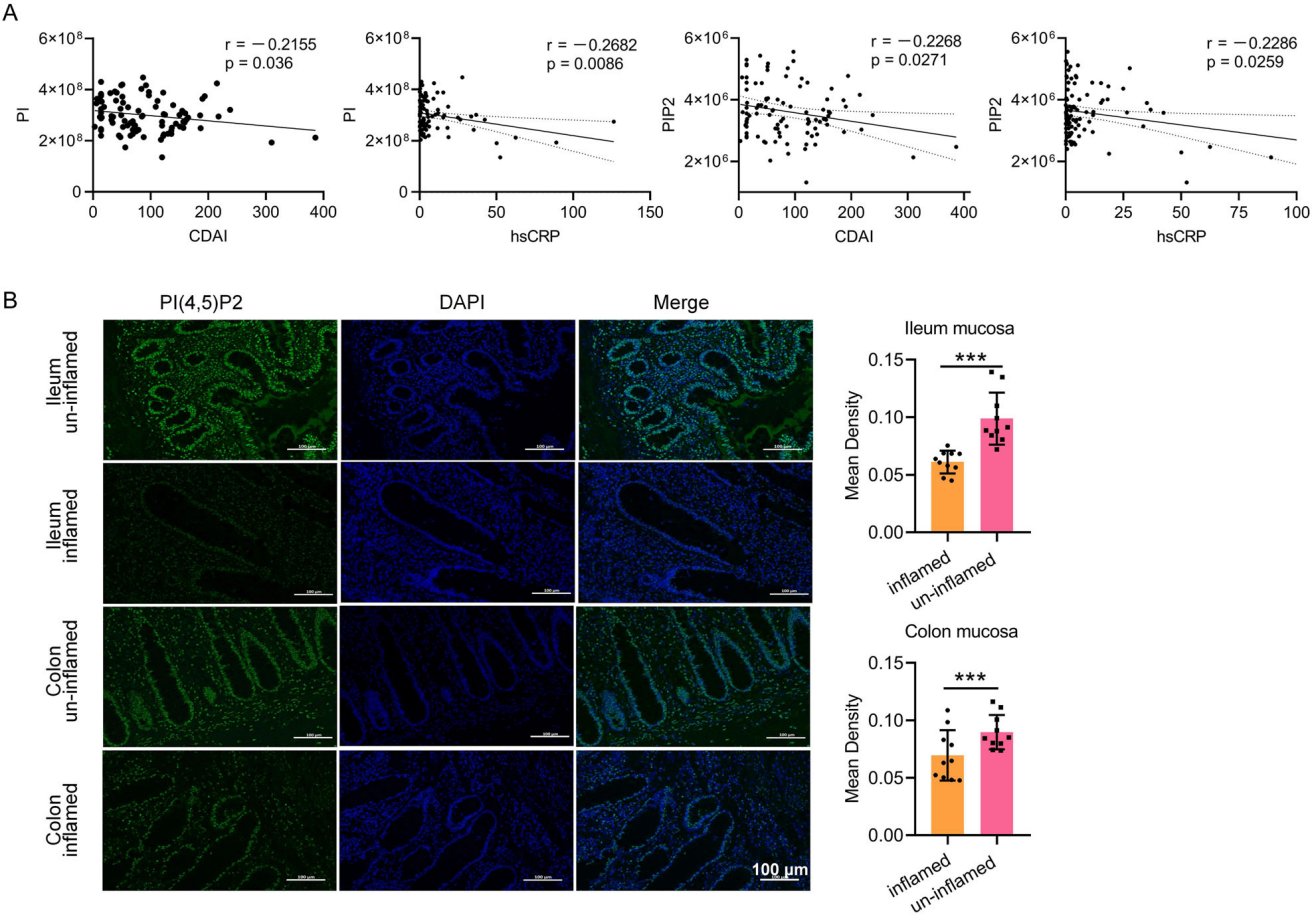
The correlation between PI, PIP2 and clinical indicators as well as PI(4,5)P2 expression. **A** Plasma PI and PIP2 levels negatively correlated with the disease activity of CD. *n* = 102. **B** PI(4,5)P2 expression was detected by IF and declined in the mucosa with inflammation lesions in patients with CD. Scale bar, 100 μm; *n* = 10. *** *P* < 0.001.

Analysis of plasma PI and PIP2 levels in relation to inflammatory markers, including CDAI and high-sensitivity C-reactive protein (hs-CRP), demonstrated a significant inverse association between PI/PIP2 and both CDAI and hs-CRP (Fig. 1A). These results indicate a negative correlation between PI/PIP2 levels and clinical disease activity in patients with CD.

To further assess PI(4,5)P2 expression, mucosal samples from patients with CD were evaluated using ten matched surgical specimens. PI(4,5)P2 expression was notably lower in inflamed colonic mucosa than in adjacent non-inflamed tissue (Fig. 1B), with similar patterns observed in ileal mucosa samples. Overall, reduced PI(4,5)P2 expression in inflamed ileocolonic mucosa suggests its potential involvement in CD pathogenesis.

### PI(4,5)P2 treatment alleviates DSS-induced colitis in mice

**Fig. 2:**
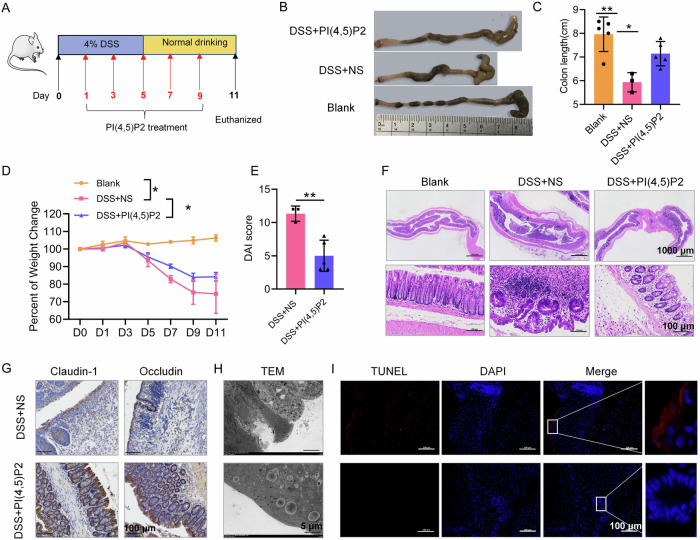
PI (4,5) P2 treatment alleviated DSS-induced colitis in mice. **A** Time flowchart of DSS-induced mice and PI(4,5)P2 treatment. **B** Representative image of the colon in each group. *n* = 5. **C** The colon length of mice in each group. **D** The change in body weight of mice in each group. **E** DAI scores of colon in each group. **F** Colonic pathology was evaluated using H&E staining. **G** The expression of intestinal epithelial barrier-related proteins such as claudin-1 and occludin was measured by IHC. Scale bar, 100 μm. **H** Ultrastructural changes in the colonic mucosa were observed under a TEM. Abbreviations of ultrastructures are labeled: Mv (microvilli), TJ (tight junction), De (desmosomes), N (nucleus), M (mitochondria), RER (rough endoplasmic reticulum), Go (Golgi body), ASS (autolysosome), and LD (Lipid droplet). Scale bar, 5 μm. **I** IECs death was measured by TUNEL staining. Scale bar, 100 μm. *P < 0.05, **P < 0.01, ***P < 0.001.

Mice with DSS-induced colitis were administered oral PI(4,5)P2 (100 μmol/L), high-dose PI(4,5)P2 enema (200 μmol/L), or low-dose PI(4,5)P2 enema (100 μmol/L), to determine the optimal concentration and administration route for PI(4,5)P2 treatment. Fig. 2A presents the time flowchart for DSS-induced mice and PI(4,5)P2 treatment. Oral or enema PI(4,5)P2 treatments improved the DAI and macroscopic and microscopic features in colitis mice (Supplementary Fig. 1A–D). While both administration routes reduced peripheral blood neutrophil proportions, significant changes were observed exclusively in the low-dose enema group (Supplementary Fig. 1E), suggesting that a 100 μmol/L PI(4,5)P2 enema may effectively mitigate acute inflammation in this model. Hence, the low-dose PI(4,5)P2 enema was chosen for subsequent experiments.

After 24 h of DSS treatment, all DSS-induced colitis mice exhibited pronounced symptoms, including bloody and sticky, loose, or watery stools, with two fatalities in the DSS + NS group. Compared to the blank group, a significant reduction in colon length was observed in the DSS groups, however, PI(4,5)P2 significantly counteracted this effect (Fig. 2B, C). While mice in the control group displayed gradual weight gain, those with DSS-induced chronic colitis experienced substantial weight loss, which was notably reduced in the PI(4,5)P2-treated group relative to the DSS + NS group (Fig. 2D). Overall, PI(4,5)P2 treatment significantly reduced DAI scores (Fig. 2E). PI(4,5)P2 improved survival rate, colon length, weight loss, and DAI in mice with DSS-induced colitis.

The inhibitory effects of PI(4,5)P2 on DSS-induced colonic pathology and inflammatory cell infiltration were confirmed through H&E staining, where PI(4,5)P2 substantially reduced colonic mucosal injury, inflammation, inflammatory cell infiltration, and crypt damage compared to the DSS + NS group (Fig. 2F). These results indicate that a 100 μmol/L PI(4,5)P2 enema effectively mitigates histological inflammation in this mouse model.

Given that IEC barrier dysfunction is a hallmark of CD, PI(4,5)P2 enema’s impact on the intestinal barrier was assessed. Treatment with PI(4,5)P2 enhanced the expression of barrier-associated proteins, including claudin-1 and occludin, in colonic tissue relative to the DSS + NS group (Fig. 2G). TEM analysis revealed severe colonic epithelial cell damage in the DSS + NS group, characterized by reduced numbers and widening of tight junctions, as well as a deficiency in intermediate junctions and desmosomes (Fig. 2H). PI(4,5)P2 enema alleviated IEC damage, as shown by an increased presence of microvilli, tight junctions, intermediate junctions, and desmosomes (Fig. 2H).

TUNEL staining indicated that significantly fewer TUNEL-positive cells were present in the colon of the DSS + PI(4,5)P2 group than in the DSS + NS group (Fig. 2I). These data suggested that the PI(4,5)P2 enema could ameliorate the mucosal barrier damage in CD mice by improving epithelial tight junction structure and reducing cell death.

### PI(4,5)P2 promotes IEC proliferation and inhibits death

**Fig. 3:**
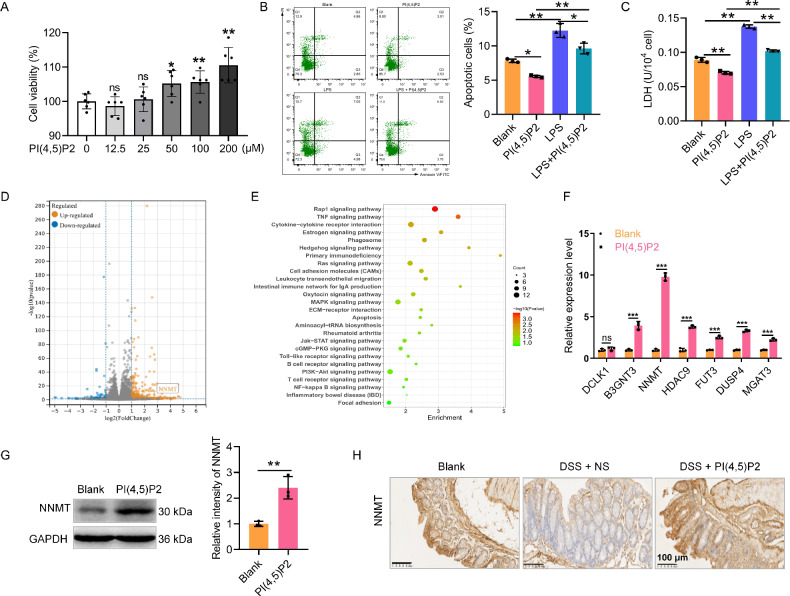
PI(4,5)P2 promotes IEC proliferation, inhibits death, and upregulates NNMT expression. **A** The viability of LPS-induced Caco-2 cells was detected by CCK8 upon PI(4,5)P2 treatment at the concentrations of 0, 12.5, 25, 50, 100, and 200 μM. **B** Dead cells were determined by flow cytometry. **C** The release of LDH level was measured by performing LDH assays. **D** The volcano plot of differentially expressed genes (DEGs) in blank and PI(4,5)P2-treated Caco-2 cells. **E** The bubble plot of the top 26 of KEGG pathway enrichment. **F** Seven DEGs with large fold changes, high expression, and upregulated in the PI(4,5)P2 group were selected for qRT-PCR verification. **G** The protein expression of NNMT in blank and PI(4,5)P2-treated Caco-2 cells was detected by WB. **H** The protein expression of NNMT in the colon mucosa of mice after PI(4,5)P2 treatment was detected by performing IHC. Scale bar, 100 μm. ns *P* > 0.05, ** *P* < 0.01, *** *P* < 0.001.

After 24 h of PI(4,5)P2 stimulation at concentrations of 50, 100, and 200 μM, viability in LPS-induced Caco-2 cells significantly increased (Fig. 3A). With cell viability maximized at 200 μM PI(4,5)P2, this concentration was utilized in all subsequent in vitro experiments. Flow cytometry analysis revealed elevated cell death in the LPS group, which was notably reduced with PI(4,5)P2 treatment relative to the blank group (Fig. 3B). Co-treatment with PI(4,5)P2 markedly mitigated LPS-induced cell death (Fig. 3B), a finding corroborated by the LDH assay (Fig. 3C). Collectively, PI(4,5)P2 may promote IEC proliferation and inhibit death under LPS exposure.

### Transcriptome sequencing reveals that PI(4,5)P2 upregulates NNMT expression in IECs

To explore the molecular mechanisms by which PI(4,5)P2 inhibits IEC death, transcriptome sequencing was performed on Caco-2 cells treated with PI(4,5)P2 compared to controls. Expression of 543 genes varied significantly following PI(4,5)P2 exposure, with 90 downregulated and 453 upregulated in the PI(4,5)P2 group (Fig. 3D). Kyoto Encyclopedia of Genes and Genomes (KEGG) pathway analysis revealed primary enrichment of DEGs in pathways such as Rap1, MAPK, Ras, PI3K-AKT, and NF-κB (Fig. 3E).

To confirm the transcriptome findings, seven DEGs with substantial fold changes and high expression levels, upregulated by PI(4,5)P2, were selected for qRT-PCR validation. NNMT, an enzyme regulating epigenetic modifications, exhibited the largest expression difference in qRT-PCR (Fig. 3F). Western blotting further confirmed that PI(4,5)P2 significantly promoted NNMT expression in Caco-2 cells (Fig. 3G). In DSS-induced colitis mice, NNMT expression in colonic mucosa was reduced relative to controls but increased following PI(4,5)P2 treatment (Fig. 3H). This suggested that PI(4,5)P2 promotes NNMT expression, and that PI(4,5)P2 function in murine colitis may be related to NNMT upregulation.

### NNMT regulates the function of PI(4,5)P2 in LPS-induced Caco-2 cells

**Fig. 4:**
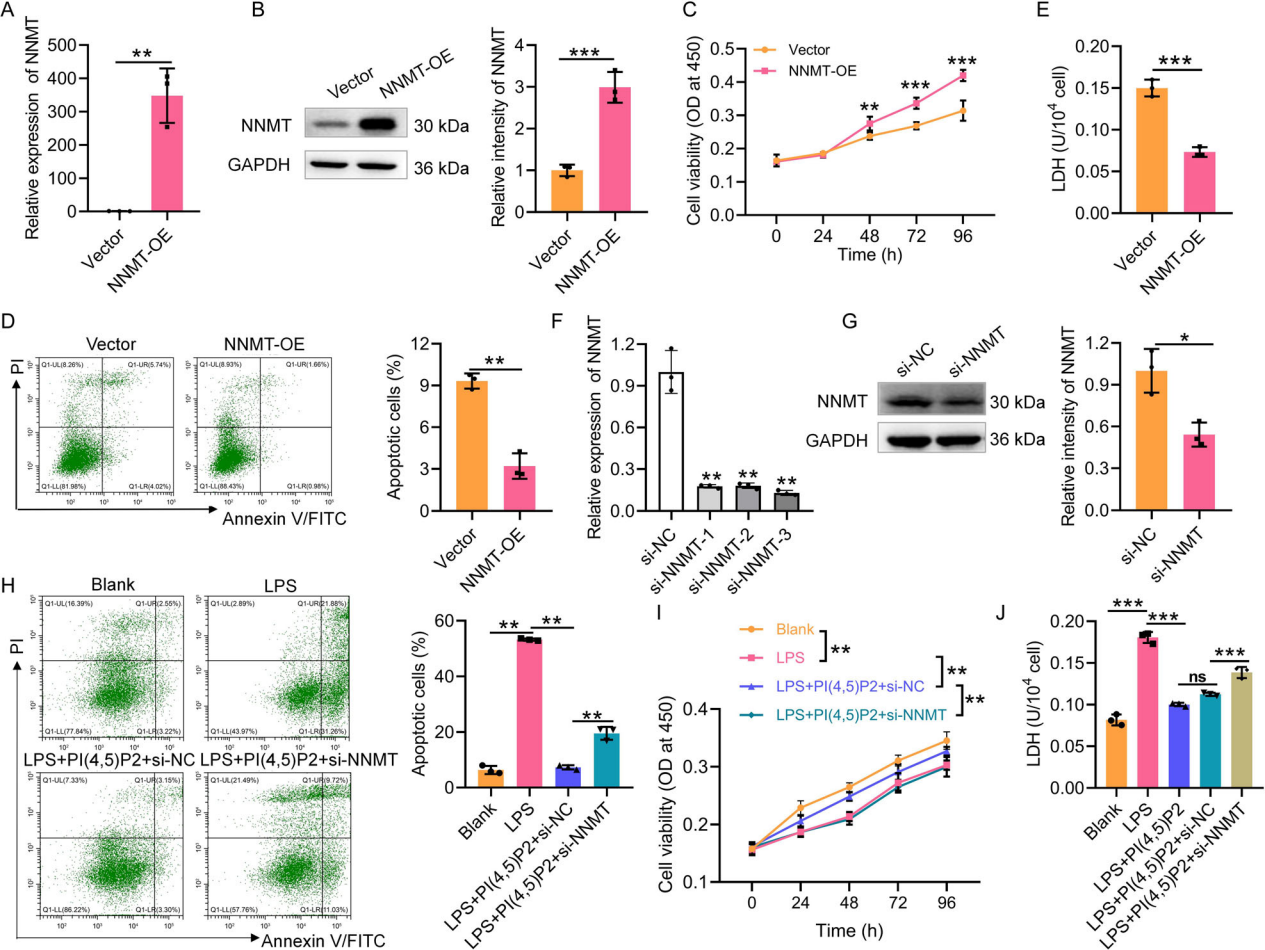
NNMT regulates the function of PI(4,5)P2 in LPS-induced Caco-2 cells. NNMT overexpression efficiency was assessed by qRT-PCR (**A**) and WB (**B**). After NNMT overexpression, the viability (**C**), cell death (**D**), and LDH level (**E**) of Caco-2 cells were detected by CCK8, flow cytometry, and LDH assay, respectively. NNMT knockdown efficiency was assessed by qRT-PCR (**F**) and WB (**G**). After NNMT interference, cell death (**H**), the viability (**I**), and LDH level (**J**) of LPS-induced Caco-2 cells upon PI(4,5)P2 treatment were detected by CCK8, flow cytometry, and LDH assay, respectively. ns *P* > 0.05, *P < 0.05, **P < 0.01, ***P < 0.001.

To further explore whether NNMT regulates PI(4,5)P2 function in IECs, we transfected the pcDNA3.1-NNMT vector into Caco-2 cells to overexpress NNMT (Fig. 4A, B). CCK8 revealed that NNMT overexpression promotes Caco-2 cell viability compared to the vector control group (Fig. 4C), while inhibiting cell death (Fig. 4D) and LDH release (Fig. 4E). Subsequently, PI(4,5)P2 was administered under LPS exposure following NNMT silencing. The knockdown of NNMT by siRNA was confirmed via qRT-PCR (Fig. 4F) and western blotting (Fig. 4G). Compared with the LPS + PI(4,5)P2+si-NC group, the NNMT knockdown group showed a significant increase in cell death (Fig. 4H) and a decrease in cell viability (Fig. 4I). Based on the LDH release results, NNMT knockdown could block the protective effect of PI(4,5)P2 on Caco-2 cells (Fig. 4J). Collectively, these results indicated that PI(4,5)P2 function in IECs was regulated by NNMT expression.

### NNMT suppresses RBP4 expression and stability by inhibiting m6A modification

**Fig. 5:**
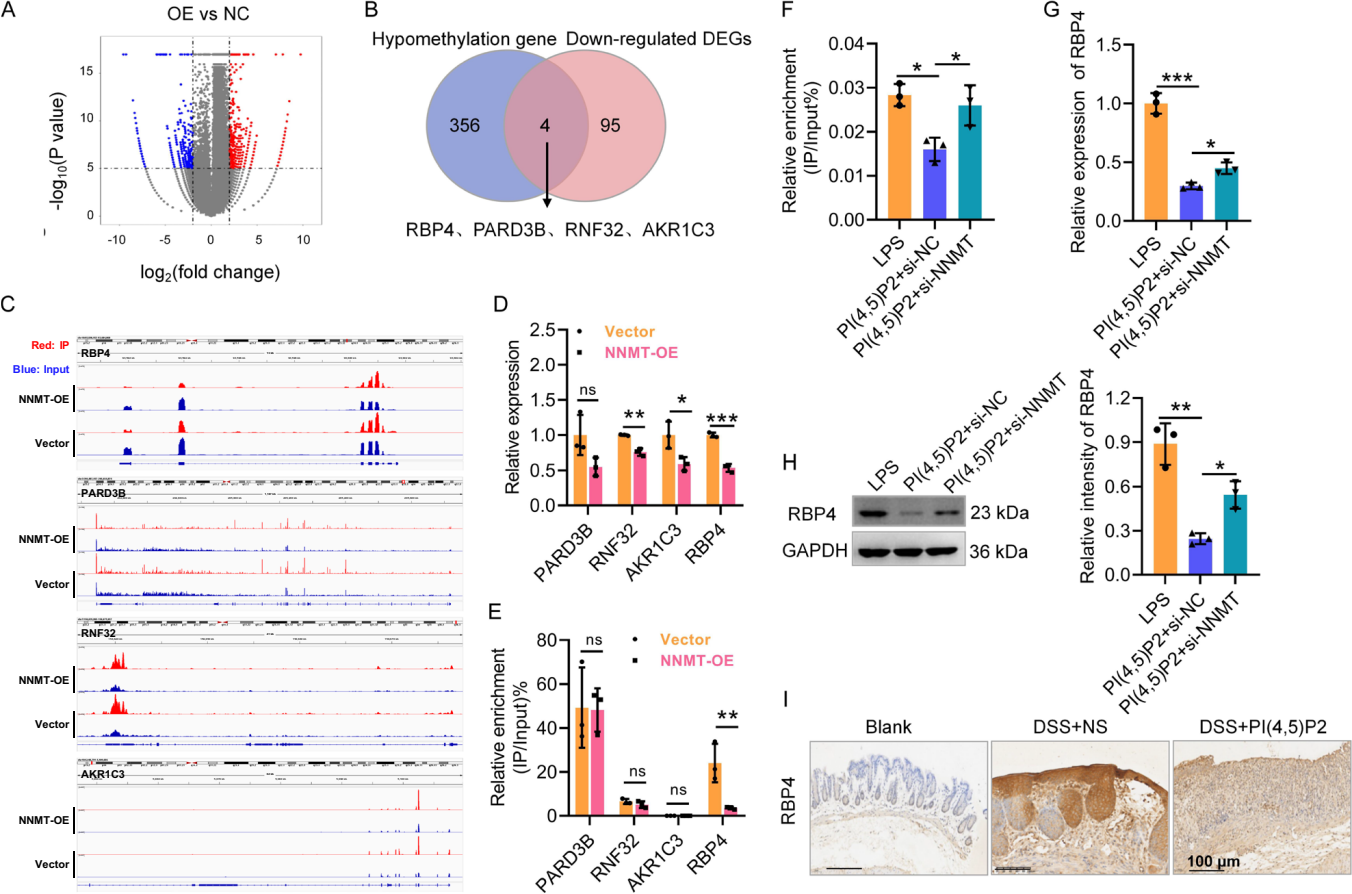
NNMT suppresses RBP4 expression and stability by inhibiting the m6A modification. **A** The volcano plot of differentially methylated genes in Caco-2 cells. **B** Conjoint analysis of hypomethylation gene in the m6A methylome from NNMT-overexpressed Caco-2 cells and down-regulated DEGs in transcriptome from input. **C** The distribution of m6A peaks of four candidate genes. **D** The mRNA expression levels of four genes in Caco-2 cells after NNMT overexpression were detected by qRT-PCR. **E** The m6A levels of four genes in Caco-2 cells after NNMT overexpression were detected by MeRIP-qPCR. **F** The m6A level of RBP4 in PI(4,5)P2-treated Caco-2 cells after NNMT knockdown was detected by MeRIP-qPCR. **G** RBP4 mRNA expression in PI(4,5)P2-treated Caco-2 cells after NNMT knockdown was detected by qRT-PCR. **H** RBP4 protein expression in PI(4,5)P2-treated Caco-2 cells after NNMT knockdown was detected by WB. **I** The protein expression of RBP4 in the colon mucosa of mice after PI(4,5)P2 treatment was detected by IHC. Scale bar, 100 μm. ns *P* > 0.05, *P < 0.05, **P < 0.01, ***P < 0.001.

NNMT, a member of the N-methyltransferase family, modulates methyl donor levels [18], and multiple N6-methyladenosine (m6A) modifications associated with CD have been identified [19]. We applied MeRIP sequencing to Caco-2 cells following NNMT overexpression to screen the downstream genes of NNMT. The m6A peak was distributed primarily in the coding sequence region (Supplementary Fig. 2A, B). A total of 720 differentially methylated genes were identified, comprising 360 hypomethylated and 360 hypermethylated genes in the NNMT-overexpression group compared to controls (Fig. 5A). KEGG pathway analysis indicated that hypomethylated genes were significantly enriched in MAPK, GnRH, Ras, and Rap1 signaling pathways (Supplementary Fig. 2C). Integrated MeRIP sequencing and transcriptomic data from the input revealed that four genes (*RBP4, PARD3B, RNF32* and *AKR1C3*) were downregulated and hypomethylated in the NNMT-overexpression group (Fig. 5B, C). Among these, *RBP4* exhibited the most substantial downregulation (Fig. 5D). MeRIP-qPCR showed that the m6A level of *RBP4* in the NNMT-overexpression group was significantly reduced compared with those in the vector control group, while there was no significant difference in the m6A levels of *PARD3B*, *RNF32*, and *AKR1C3* (Fig. 5E). Hence, we focused on *RBP4* in subsequent analyses.

PI(4,5)P2 intervention decreased the m6A level of *RBP4* in LPS-exposed Caco-2 cells, while NNMT knockdown restored the reduction in *RBP4* m6A modification induced by PI(4,5)P2 (Fig. 5F). Additionally, NNMT knockdown partially restored RBP4 mRNA (Fig. 5G) and protein (Fig. 5H) expression levels, which PI(4,5)P2 had otherwise suppressed. In contrast to NNMT expression (Fig. 3H), RBP4 expression in the colonic mucosa of DSS-induced colitis mice decreased following PI(4,5)P2 intervention (Fig. 5I). Overall, *RBP4* acts as a key downstream gene of NNMT in response to PI(4,5)P2.

**Fig. 6:**
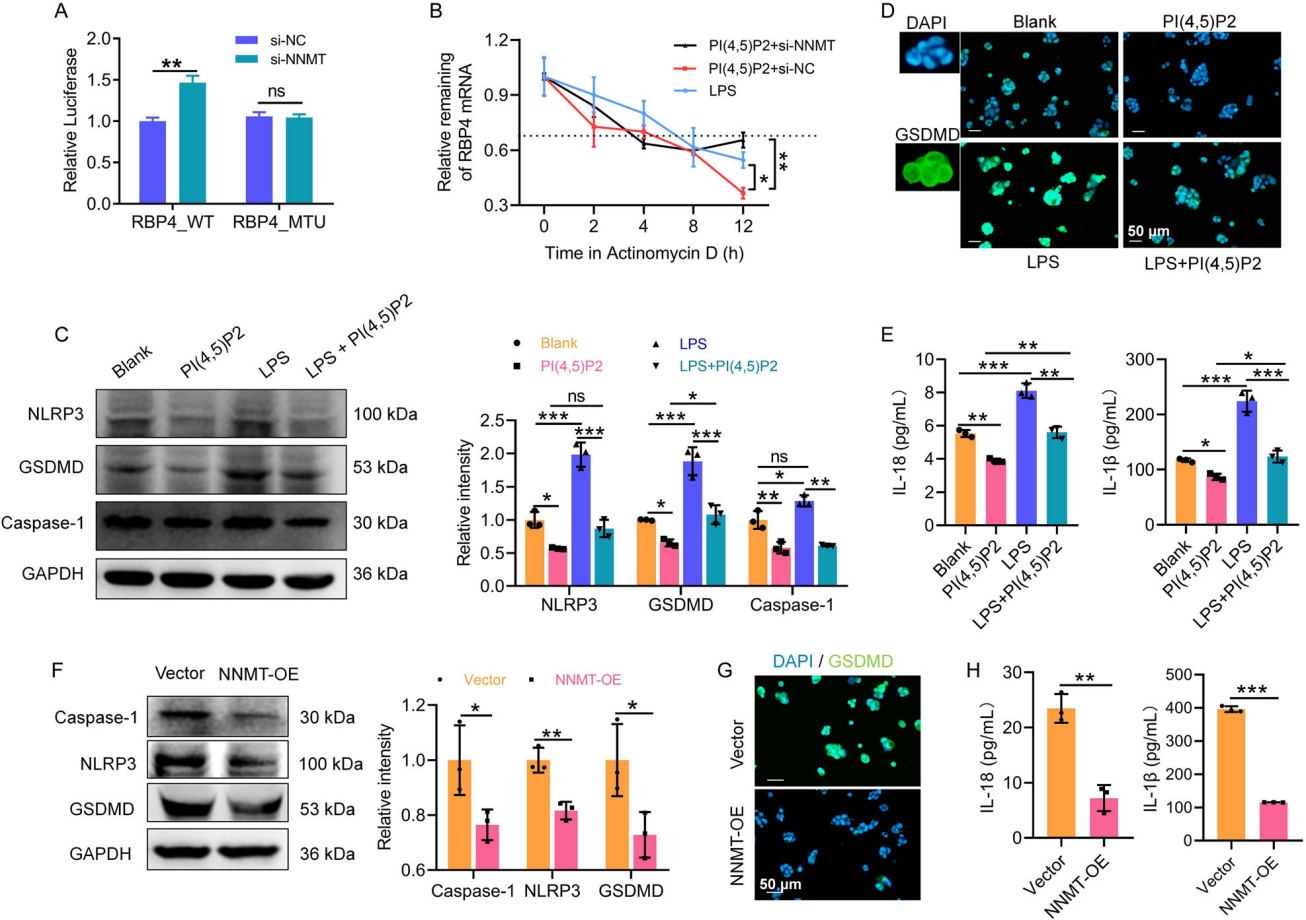
PI(4,5)P2 and NNMT inhibited pyroptosis in LPS-induced Caco-2 cells. Continuation of Fig. 5, to identify NNMT suppresses RBP4 expression and stability by promoting the m6A modification, the binding between NNMT and RBP4 was evaluated by dual-luciferase reporter assay after mutant m6A sites in RBP4 3′ UTR sequence (**A**) and after actinomycin D treatment, the RBP4 mRNA expression in PI(4,5)P2-treated Caco-2 cells after NNMT knockdown was detected by qRT-PCR (**B**). **C** The protein expression of caspase-1, GSDMD, and NLRP3 in Caco-2 cells was detected by WB upon PI(4,5)P2 treatment. **D** The protein expression of GSDMD in Caco-2 cells was stained by IF upon PI(4,5)P2 treatment. Scale bar, 50 μm. **E** The concentration of IL-18 and IL-1β was detected by ELISA upon PI(4,5)P2 treatment. After NNMT overexpression, the protein expression of caspase-1, GSDMD, and NLRP3 (**F**), anti-GSDMD staining (**G**), and IL-18 and IL-1β concentration (**H**) of Caco-2 cells were detected by WB, IF, and ELISA, respectively. *P < 0.05, **P < 0.01, ***P < 0.001.

To determine whether NNMT regulates *RBP4* mRNA expression in an m6A-dependent manner, we established dual-luciferase reporter plasmids containing wild-type (RBP4_WT) or mutant (RBP4_MT) m6A sites in the *RBP4* 3′-UTR sequence (Supplementary Fig. 2D). NNMT knockdown significantly increased the relative luciferase activities of the reporters with wild-type m6A sites; while this effect was abolished in reporters containing mutated m6A sites (Fig. 6A).

The actinomycin D experiment showed that the half-life of *RBP4* mRNA was reduced in the PI(4,5)P2+si-NC group compared with that in the LPS group, and increased by PI(4,5)P2+si-NNMT co-treatment (Fig. 6B). This suggests that NNMT may reduce *RBP4* mRNA stability via m6A modification.

### PI(4,5)P2 regulates the NNMT-RBP4 pathway to inhibit IEC pyroptosis

RBP4, a circulating adipokine [20], exerts pro-inflammatory effects by activating the NLRP3 inflammasome and is implicated in GSDMD-dependent pyroptosis [21, 22]. As a component of the cell membrane, PI(4,5)P2 interacts with the N-terminus of GSDMD to form transmembrane pores during pyroptosis [23]. Furthermore, we found that PI(4,5)P2 protects the intestinal barrier in CD mice by inhibiting Caco-2 cell death. KEGG pathway analysis indicated that DEGs after PI(4,5)P2 intervention were mainly involved in pyroptosis-related signaling pathways. Accordingly, we further explored PI(4,5)P2 function in Caco-2 cell pyroptosis.

In LPS-exposed Caco-2 cells, western blot analysis showed that PI(4,5)P2 treatment significantly downregulated NLRP3, GSDMD, and caspase-1 expression compared to the blank group, counteracting the pyroptotic effects of LPS (Fig. 6C). IF analysis further supported PI(4,5)P2’s inhibition of LPS-induced GSDMD upregulation (Fig. 6D). Additionally, PI(4,5)P2 treatment significantly lowered levels of pyroptosis-related cytokines IL-18 and IL-1β in LPS-exposed Caco-2 cells (Fig. 6E). Collectively, PI(4,5)P2 inhibited IEC pyroptosis under LPS exposure.

**Fig. 7:**
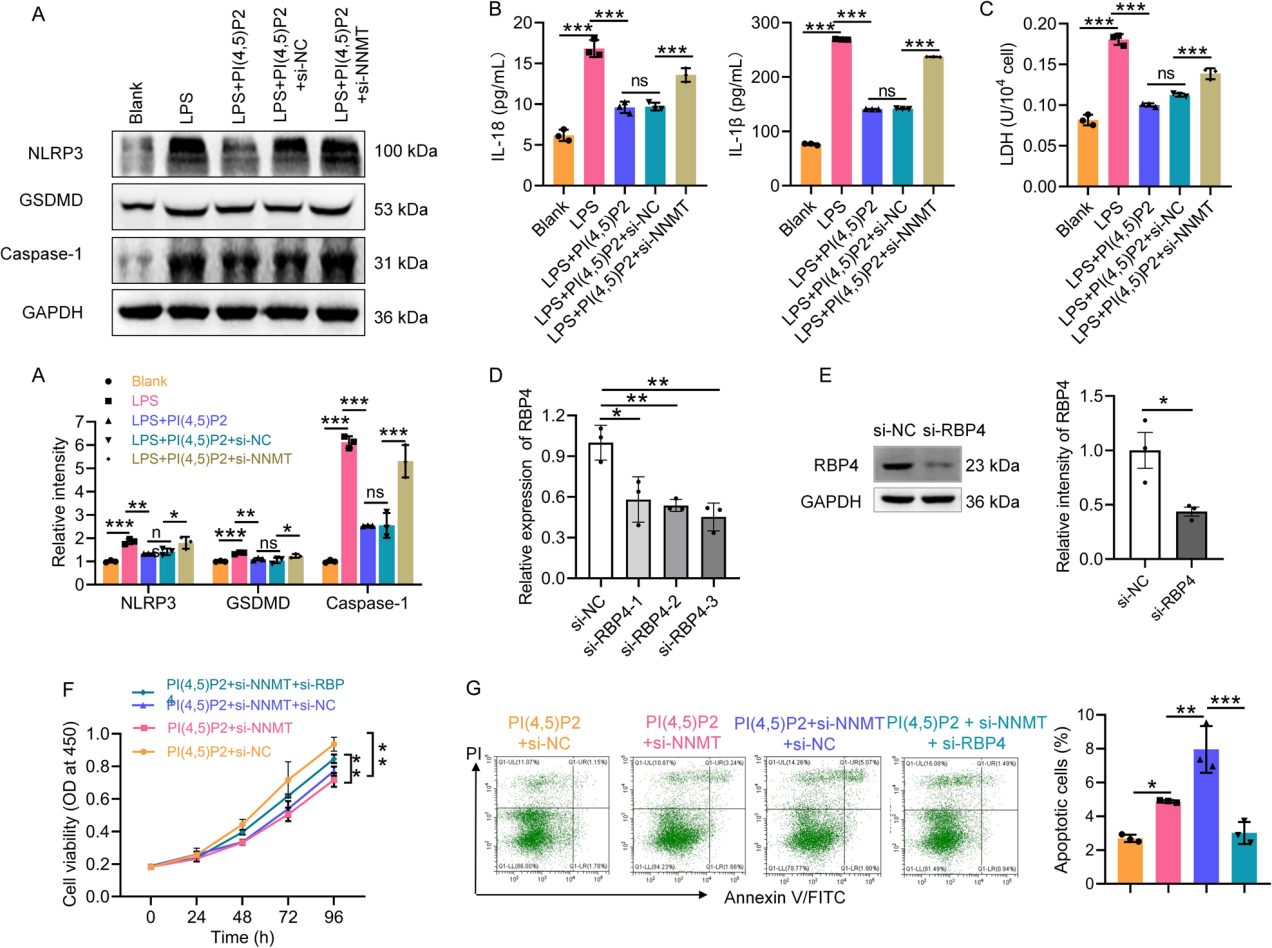
PI(4,5)P2 regulates the NNMT-RBP4 pathway to inhibit IEC pyroptosis. To identify PI(4,5)P2 suppresses pyroptosis in IECs depending on NNMT expression, after NNMT know down, the protein expression of caspase-1, GSDMD, and NLRP3 (**A**), IL-18 and IL-1β concentration (**B**), and LDH level (**C**) of LPS-induced Caco-2 cells upon PI(4,5)P2 treatment were detected by WB, ELISA, and LDH assay, respectively. RBP4 knockdown efficiency was assessed by qRT-PCR (**D**) and WB (**E**). Simultaneous transfection of si-NNMT and si-RBP4 into the PI(4,5)P2-treated Caco-2 cells, followed by detection of viability (**F**) and cell death (**G**) was detected by CCK8 and flow cytometry, respectively. ns *P* > 0.05, *P < 0.05, **P < 0.01, ***P < 0.001.

NNMT overexpression further suppressed pyroptosis in Caco-2 cells, evidenced by reduced protein levels of caspase-1, GSDMD, and NLRP3 following pcDNA3.1-NNMT transfection (Fig. 6F). IF analysis confirmed that NNMT overexpression downregulated GSDMD expression (Fig. 6G). IL-18 and IL-1β concentrations in the cell supernatant were significantly reduced following NNMT overexpression (Fig. 6H). In contrast, NNMT knockdown reversed the PI(4,5)P2-mediated reduction in caspase-1, GSDMD, and NLRP3 expression (Fig. 7A), as well as IL-18 and IL-1β release (Fig. 7B). LDH release results confirmed that NNMT knockdown blocked the protective effect of PI(4,5)P2 on Caco-2 cells (Fig. 7C). Collectively, these results indicated that PI(4,5)P2 suppresses IEC pyroptosis by upregulating NNMT expression.

**Fig. 8:**
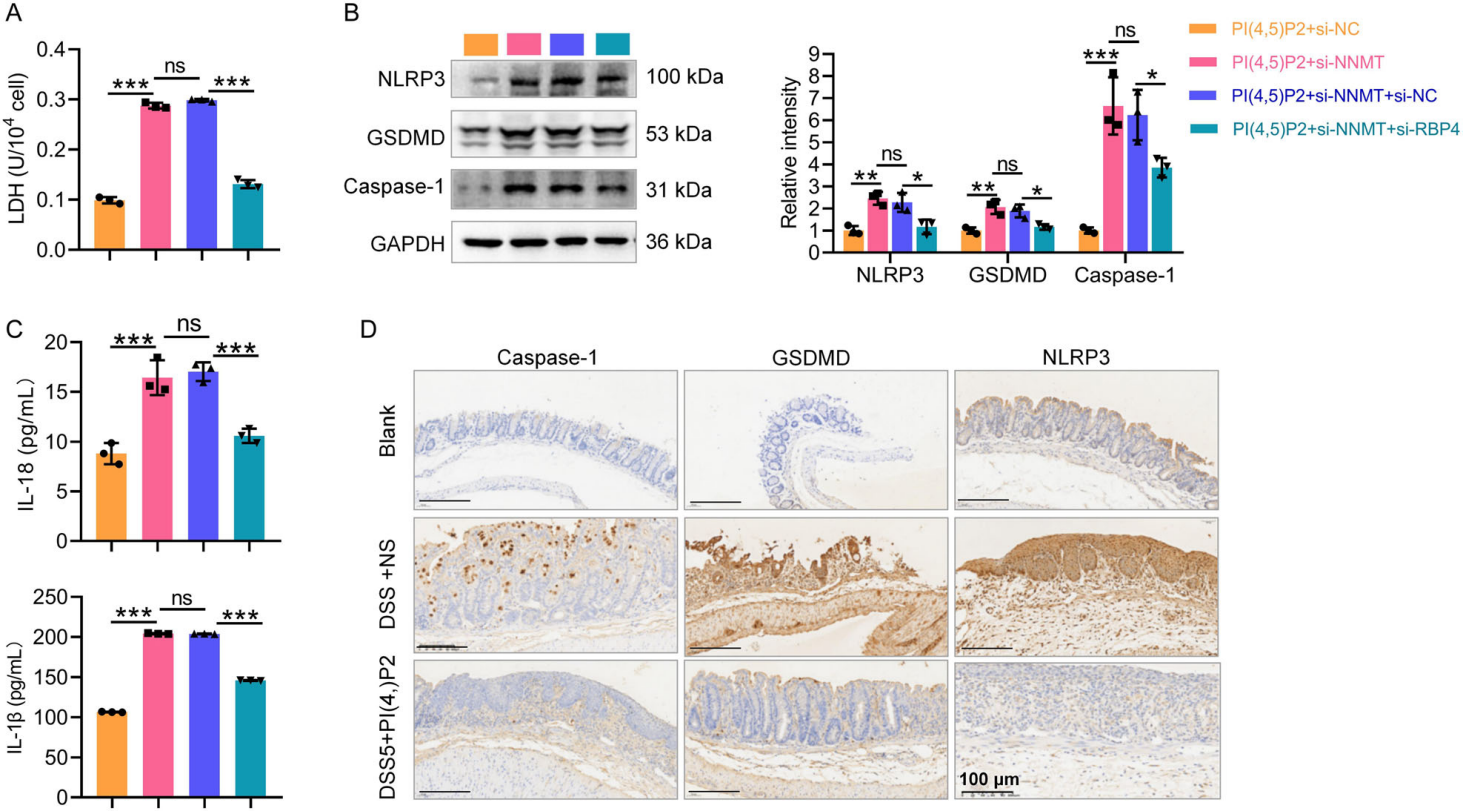
Continuation of Fig. 7, to identify PI(4,5)P2 regulates the NNMT-RBP4 pathway to inhibit IEC pyroptosis. Simultaneous transfection of si-NNMT and si-RBP4 into the PI(4,5)P2-treated Caco-2 cells, and then the LDH level (**A**), protein expression levels of caspase-1, GSDMD, and NLRP3 (**B**), and IL-18 and IL-1β concentration (**C**) were detected by LDH assay, WB, and ELISA, respectively. **D** The expression of pyroptosis markers including caspase-1, GSDMD, and NLRP3 in the mucosa of CD mice after PI(4,5)P2 treatment was detected using IHC. Scale bar, 100 μm. ns *P* > 0.05, *P < 0.05, **P < 0.01, ***P < 0.001.

To investigate whether the PI(4,5)P2-induced upregulation of NNMT and inhibition of pyroptosis depends on RBP4, Caco-2 cells were transfected with siRNAs targeting NNMT and RBP4 under LPS exposure and PI(4,5)P2 treatment. siRNA successfully inhibited RBP4 at both the mRNA (Fig. 7D) and protein levels (Fig. 7E). CCK8 assays revealed that the reduction in cell proliferation due to NNMT knockdown was partially reversed by RBP4 knockdown (Fig. 7F). According to flow cytometry analysis, the PI(4,5)P2+si-NNMT group exhibited higher cell death than the PI(4,5)P2+si-NC group; however, si-RBP4 partially reversed this effect (Fig. 7G). The LDH release assay confirmed these observations (Fig. 8A). Moreover, RBP4 knockdown counteracted the si-NNMT-induced upregulation of NLRP3, GSDMD, and caspase-1 expression (Fig. 8B), as well as IL-1β and IL-18 levels (Fig. 8C) in PI(4,5)P2-treated Caco-2 cells. These results suggest that PI(4,5)P2/NNMT may regulate IEC pyroptosis by modulating RBP4 mRNA stability.

To assess whether PI(4,5)P2 inhibits pyroptosis in DSS-induced colitis, pyroptosis marker expression in the colonic mucosa of CD mice was examined using IHC. Caspase-1, GSDMD, and NLRP3 levels were elevated in the DSS + NS group compared to the control, indicating active pyroptosis in CD progression. PI(4,5)P2 enema treatment significantly decreased the expression of caspase-1, GSDMD, and NLRP3 (Fig. 8D), suggesting that PI(4,5)P2 inhibits pyroptosis in the intestinal mucosa of colitis mice.

We further collected the inflammatory sites from the intestinal surgical specimens of 21 patients with CD and subjected them to IF and FISH analyses. A positive correlation was observed between PI(4,5)P2 and NNMT protein expression (Supplementary Fig. 3A), as well as between GSDMD and RBP4 protein abundance (Supplementary Fig. 3B). Meanwhile, RBP4 mRNA and NNMT protein were negatively correlated (Supplementary Fig. 3C). These results indicated significant relationships between PI(4,5)P2, NNMT, RBP4, and GSDMD in patients with CD.

## Discussion

Reportedly, abnormal lipid metabolism is closely related to CD pathogenesis [24–26]. In a previous study, we showed that PI and its derivative PIP2 may be key regulatory factors. In the current study, we confirmed that plasma levels of PI and PIP2 correlate with active CD in patients, with a marked reduction of PI(4,5)P2 observed in the inflamed intestinal mucosa. Mechanistically, PI(4,5)P2 upregulates NNMT expression to suppress pyroptosis in IECs by decreasing *RBP4* mRNA stability via m6A modification. This PI(4,5)P2/NNMT/RBP4/pyroptosis axis may represent a promising therapeutic target for CD treatment.

PI(4,5)P2, a plasma membrane signaling molecule, is critical for immune-inflammatory processes [27]. Reduced PI(4,5)P2 synthesis mediated by ADP-ribosylation factor 3 (ARF3) inactivation can suppress pro-inflammatory responses of LPS-induced macrophages [28]. Gangliosides induce inflammatory responses by regulating phosphatidylinositol 4-phosphate 5-kinase alpha (PIP5Kα) and PI(4,5)P2 in astrocytes [29], while reorganizing PI(4,5)P2 with n-3 polyunsaturated fatty acids may suppress CD4( + ) T cell proliferation [30]. Collectively, these data suggest that PI(4,5)P2 contributes to the regulation of immune-inflammatory diseases. As an immune-mediated disease, inflammatory cell imbalances and intestinal barrier injury were the key features of CD. In a previous small-sample study, we detected elevated levels of PI and PIP2 in the plasma of patients with CD, and this finding was confirmed in the present study that involved a large cohort. Consistent with these findings, we found that PI(4,5)P2 was downregulated in inflamed colonic and ileal mucosa of patients with CD, suggesting that PI(4,5)P2 may be a protective factor in CD.

To explore the underlying mechanisms, in vivo cell and in vitro colitis models were applied. PI(4,5)P2 alleviated colitis in the DSS-induced mouse colitis model, improving survival rates, colon lengths, weight loss, and DAI scores. Notably, the protective effect of PI(4,5)P2 was associated with reduced cell death in the colonic mucosa. The LPS-induced Caco-2 cell model also showed reduced cell death following PI(4,5)P2 treatment. Exploring how PI(4,5)P2 inhibited IEC death may facilitate the identification of a new therapy for CD.

Pyroptosis is a pro-inflammatory programmed cell death, triggered primarily by the NLRP3 inflammasome and mainly requires the cleavage of GSDMD into its N- and C-termini by caspase-1 to release cytokines, including IL-1β and IL-18 [31]. Thus, pyroptosis plays a vital role in mucosal immune response and gut homeostasis in CD [32]. Inhibiting pyroptosis in IECs can effectively improve intestinal inflammation, reduce epithelial cell death, and promote intestinal mucosal barrier recovery [33]. For example, the artemisinin analogue, SM934, was shown to afford protection against intestinal barrier disruption and inflammatory progression by inhibiting caspase-1-mediated pyroptosis in epithelial cells [34]. GSDME-mediated pyroptosis reportedly promotes intestinal inflammation progression in CD by releasing pro-inflammatory cytokines, whereas its knockdown in IECs can protect from trinitro-benzene-sulfonic acid-induced colitis in mice [35]. Likewise, the selective caspase-1 inhibitor VX765 alleviates DSS-induced intestinal barrier dysfunction and inflammation by inhibiting pyroptosis in IECs [36]. Therefore, inhibition of pyroptosis in IECs shows potential as a new strategy that may promote inflammation resolution and mucosal healing in CD. PI(4,5)P2 in the cell membrane reportedly binds to the N-terminus of GSDMD to form transmembrane pores involved in pyroptosis [23]. Our results showed that PI(4,5)P2 effectively inhibits pyroptosis in LPS-induced Caco-2 cells and the colonic mucosa of mice with colitis. Therefore, PI(4,5)P2 not only acts as a key lipid molecule in pyroptosis but also alleviates CD by inhibiting pyroptosis in IECs.

In the present study, knocking down NNMT significantly abolishes PI(4,5)P2-mediated suppression of pyroptosis. Thus, PI(4,5)P2 may inhibit intestinal pyroptosis via NNMT. NNMT belongs to the family of N-methyltransferases and functions primarily in the transfer of methyl group from S-adenosylmethionine (SAM) to nicotinamide (NA) to generate S-adenosine hypercysteine and 1-methylnicotinamide [18]. SAM is a universal methyl donor for histone proteins, non-histone proteins, DNA, and RNA [18]; hence, NNMT can regulate the methylation modification of downstream genes. However, there are no reports of NNMT regulating m6A methylation modification of target genes. The m6A is a common modification of mRNA that plays an important role in various cellular functions and diseases, including pyroptosis and CD. For instance, m6A may mediate the mucosal immune-inflammatory microenvironment of CD and the therapeutic response against TNF [37]. Animal studies have confirmed that overexpression of the m6A methyltransferase, METTL3, aggravates LPS-induced inflammation of IECs and DSS-induced colitis in mice [38]. Similarly, METTL14 deficiency leads to stem cell apoptosis in the mouse colon, resulting in mucosal barrier dysfunction and severe colitis [39]. These studies prompted us to hypothesize that NNMT may inhibit pyroptosis and attenuate CD progression via m6A-modification. Our results support this hypothesis, demonstrating that NNMT inhibits RBP4 expression and stability in an m6A-dependent manner, thereby inhibiting pyroptosis in IECs.

RBP4 is a critical regulator in lipid- and metabolism-related diseases [20, 40], and two studies support our results that RBP4 is an upstream regulator of the pyroptosis signaling pathway [21, 22]. Moraes-Vieira et al. [21], found that RBP4 exerts a pro-inflammatory effect by releasing IL-1β via priming of the NLRP3 inflammasome through the TLR4/MD2 receptor complex in adipose tissues. Meanwhile, Zhang et al., suggested that RBP4 promotes cardiac injury following myocardial infarction by activating the NLRP3 inflammasome, cleaving the caspase-1 precursor, and inducing GSDMD-dependent pyroptosis [22]. These results suggest that RBP4 is an important regulatory molecule in pyroptosis. Consistent with this study, we found that knocking down RBP4 reversed the effect of NNMT knock down on pyroptosis in Caco-2 cells upon PI(4,5)P2 treatment. Further confirming this relationship, we observed a significant correlation between the expression of PI(4,5)P2 and NNMT, NNMT and RBP4, and RBP4 and GSDMD in patients with CD. In short, the PI(4,5)P2/NNMT axis downregulates RBP4 via the m6A modification to inhibit pyroptosis in CD.

Our study had some limitations. First, we performed semi-quantitative and non-targeted lipidomics to analyze plasma levels of PI and PIP2. In the future, a PI(4,5)P2 standard for lipidomic studies should be developed and targeted quantitative lipidomics of PI(4,5)P2 should be performed. A multicenter study with a larger sample size should be conducted to further clarify plasma levels of PI(4,5)P2 in patients with CD and its potential as a marker for CD diagnosis and treatment. Second, m6A methyltransferases and recognition proteins for NNMT-mediated *RBP4* m6A modification should be further explored. Third, the specific mechanism through which PI(4,5)P2 regulates NNMT remains unclear. A recent study suggested that PI(4,5)P2 potentially functions as an epigenetic regulator of rRNA gene transcription [10]. Moreover, NNMT promoter activity is upregulated by transcription factors in tumor cells [41, 42], suggesting that PI(4,5)P2 may impact NNMT expression by regulating its gene transcription or promoter activity. Additionally, given that mice fed a high-fat diet exhibit elevated NNMT expression [43], NNMT may be regulated by PI(4,5)P2 in a lipid metabolism-related manner. Hence, in-depth investigations are warranted to elucidate the mechanism through which PI(4,5)P2 regulates NNMT expression.

## Conclusion

To the best of our knowledge, the present study is the first to report that PI(4,5)P2 may protect colitis mice and IECs by suppressing pyroptosis. This effect was associated with NNMT-mediated *RBP4* m6A modification. This study provides novel insights into the protective mechanisms of PI(4,5)P2 in CD and may enable the development of novel therapies based on lipid metabolism regulation.

## Supplementary information

Supplementary Information

Original Western blot images

## Data Availability

All datasets generated and analyzed during this study are presented in this published article and its Supplementary information files. Additional data are available from the corresponding author upon reasonable request.
