# Supplementary material for: PI(4,5)P2 alleviates colitis by inhibiting intestinal epithelial cell pyroptosis through NNMT-mediated RBP4 m6A modification
Source: Cell Death Dis. 2024 Dec 20;15(12):923. doi: 10.1038/s41419-024-07276-3 (PMC11661994; doi:10.1038/s41419-024-07276-3)
Supplement: Supplementary file 1 — Supplementary Information [file 41419_2024_7276_MOESM1_ESM.pdf]

Supplementary Information

Materials and Methods

Participants and clinical sample collection

The inclusion criteria were age ≥18 years, confirmed CD based on the current guideline, and available plasma samples. The exclusion criteria comprised patients diagnosed with cancer and other immune system-related diseases, and those not followed up regularly at our inflammatory bowel disease (IBD) center.

Data on participant demographics, including family history, disease activity indices, intestinal surgery, intestinal fistula/abscess, serum inflammatory indicators, medication history, perianal disease, and disease location, were collected from the electronic database of our hospital.

Blood samples were collected from patients with CD after overnight fasting. After centrifuging, the plasma fraction was collected and stored at −80 °C for further study. Paraffinized surgical specimens from patients with CD were also collected.

PI(4,5)P2 treatment in a dextran sulfate sodium (DSS)-induced colitis model

The experimental process complied with the regulations on the Management of Experimental Animals, and ethical requirements were approved by the Ethics Committee of our hospital. DSS with a molecular weight of 5000 g/moL was dissolved in distilled water to prepare a 4% DSS solution. PI(4,5)P2 (No. P4508) was synthesized by Echelon Biosciences (UT, USA) and dissolved in sterile double-distilled water (ddH$_2$O) as a stock solution. Male C57BL/6 mice were divided into five groups (n = 5/group): Blank, DSS + normal saline (NS), PI(4,5)P2 high dose enema [DSS + H-PI(4,5)P2, concentration 200 μmol/L], PI(4,5)P2 low dose enema

[DSS + L-PI(4,5)P2, concentration 100 μmol/L], and oral (DSS + O-PI(4,5)P2, concentration 100 μmol/L). On days 0–5, all mice, excluding the blank group, were provided drinking water containing 4% DSS. The mice in the blank group consumed normal water. Furthermore, 100 μL of PI(4,5)P2 solution at different concentrations was administered every other day beginning on day 2 to the DSS + H-PI(4,5)P2 and DSS + L-PI(4,5)P2 groups; an NS enema was administered to the DSS + NS group; mice in the DSS + O-PI(4,5)P2 group received drinking water containing 100 μmol/L PI(4,5)P2. On days 6–9, the mice stopped drinking water with 4% DSS; the remaining treatment was the same as on days 0–5. The mice were sacrificed by cervical dislocation on day 11. Blood was collected from the orbit, and colon tissues were harvested. From the date of modeling, activity, eating, weight, stool blood, and stool characteristics were observed daily, and the DAI of the mice was scored. All animal experiments were performed four times: twice to determine the best concentration for intervention with PI(4,5)P2 and twice under the current condition.

Immunofluorescence (IF)

Paraffinized surgical specimens from patients with CD were deparaffinized in xylene, followed by rehydration in water using graded alcohol and incubation with antibodies against PI(4,5)P2 (1:200, Z-P045, Echelon Biosciences), RBP4 (1:200, 11774-1-AP, Proteintech), and GSDMD (1:500, 20770-1-AP, Proteintech) overnight at 4 °C. Next, the sections were incubated with an Alexa Fluor® 488-conjugated anti-rabbit secondary antibody (1:400, GB35303, Servicebio, Wuhan, China) at 25 °C and counterstained with DAPI (ab104139, Abcam, Cambridge, United Kingdom) for nuclei staining.

For cellular IF, Caco-2 cells were fixed in 4% paraformaldehyde for 20 min. To detect intracellular antigens, cells were permeabilized with Triton X-100 for 10 min, blocked

with 5% bovine serum albumin (BSA) for 60 min, and then incubated with anti-gasdermin D (GSDMD; 1:200, A20197, ABclonal, Wuhan, China) overnight at 4 °C. Alexa Fluor® 488-conjugated anti-rabbit secondary antibodies (1:300, ab150077, Abcam) were added for 1 h and DAPI staining was performed. Independent experiments were performed in triplicate.

Images were captured using a microscopic imaging system (Nikon DS-U3, Tokyo, Japan). Three random high-power fields (×200) were selected per slice to analyze the positive area and positive cumulative integrated optical density (IOD) in the region measured using Aipathwell V2 (Servicebio). Average optical density (mean density) was applied to evaluate the target protein expression, where the average optical density was the cumulative IOD/positive pixel area.

Flow cytometry

Peripheral blood of the mice was collected and centrifuged at 2500 rpm and 4 ^{0}C for 10 min. Blood cells from the lower layer were obtained and red cell lysates were added. The blood cells were treated with an Fc block (Anti-mouse CD16/32, Mouse BD Fc Block) for 15 min. To evaluate neutrophils and monocytes/macrophages, the blocked cells were stained with anti-mouse Ly6G, Ly6C, CD11b, CD54, and LRRC32 antibodies for 20–30 min. Next, cells were mixed with FACS buffer, centrifuged, and resuspended in FACS buffer containing propidium iodide. The stained cells were processed using a FACSCanto II flow cytometer (BD Biosciences), and the data were analyzed using FlowJo software (FlowJo LLC, Ashland, USA).

For cell death detection of Caco-2 cells, the Annexin V-fluorescein isothiocyanate apoptosis kit (C1062, Beyotime, China) was applied. Caco-2 cells were harvested and washed twice with cold phosphate-buffered saline. Cell pellets were resuspended in 195 μL of 1× binding buffer and incubated with 5 μL of Annexin V-fluorescein

isothiocyanate for 15 min at 25 °C in the dark. The cells were washed with 200 μL of 1× binding buffer, centrifuged, and resuspended in 190 μL of 1× binding buffer. Next, cells were incubated with 10 μL of propidium iodide. The proportion of apoptotic cells was determined by flow cytometry (FACSVerseTM, Becton, Dickinson and Company, USA). Independent experiments were performed in triplicate.

Hematoxylin and eosin (H&E) staining

Mouse tissues were fixed in 10% formalin, embedded in paraffin, cut into sections (3 μm), and stained with H&E to evaluate the pathological damage caused by colitis. The images were observed under a microscope (Nikon Eclipse Ci) equipped with an imaging system (Digital Sight DS-FI2; Eclipse C2; Nikon). The histological score was evaluated by two professional gastrointestinal pathologists. The severity/range of inflammatory infiltration and crypt damage served as score indicators. Each indicator was scored from 0 to 4 as the method used in reference 17 in the manuscript.

Immunohistochemistry (IHC)

The colonic sections from colitis mice were deparaffinized in xylene and rehydrated via a graded alcohol series. For IHC, the sections were blocked with 5% BSA and incubated with primary antibodies, namely anti-claudin-1 (1:400, GB11032, Servicebio), anti-occludin (1:500, GB11022, Servicebio), NNMT (1:150,15123-1-AP, Proteintech, China), RBP4 (1:250, 11774-1-AP, Proteintech), GSDMD (1:150, 20770-1-AP, Proteintech), caspase-1 (1:200, SC-56036, Santa Cruz, USA), and NLRP3 (0.1 μg/mL, GB114320, Servicebio), overnight at 4 °C. Sections were then incubated with horseradish peroxidase (HRP)-conjugated goat anti-rabbit IgG (1:200, GB23303, Servicebio). Images were obtained using an XSP-C204 biological microscope (COIC, China) and the NanoZoomer 2·0-HT microdissection system

(Bensons, Japan). The cumulative IOD and tissue area of the entire interface were analyzed using Aipathwell V2 (Servicebio).

Transmission electron microscopy (TEM) observation of the murine colonic ultrastructure

Fresh colon tissue was obtained immediately after euthanasia and placed in 2.5% glutaraldehyde solution at 4 °C for 2–4 h. Subsequently, post-fixation dehydration was performed. The colon tissues were incubated with propylene oxide. Small tissue pieces (1–3 mm) were embedded in paraffin and sectioned using an ultramicrotome. Next, slices were placed on copper grids and negatively stained for 15 min using 2% uranyl acetate. Ultrastructural observations were performed via TEM (HT7700; Hitachi, Tokyo, Japan).

Terminal deoxynucleotidyl transferase-mediated dUTP nick end labeling (TUNEL) assay

An *in situ* cell death detection kit was applied to assess cell death in the colon sections using the TUNEL staining solution (G1501, Servicebio), according to the manufacturer's instructions. Images were captured via fluorescence microscopy (Nikon DS-U3), and three discontinuous visual fields were selected and photographed.

Transcriptome sequencing

Caco2 cells were divided into the control group and PI(4,5)P2-treatment group. Cells in the PI(4,5)P2 group were treated with 200 μM PI(4,5)P2. Total RNA was extracted using the TRIzol method (15596018, Invitrogen, Carlsbad, USA) and quality analysis

was performed using a NanoDrop spectrophotometer (Thermo Fisher Scientific). A cDNA library was constructed using the VAHTS Universal V6 RNA-seq Library Prep Kit for Illumina (NR604; Vazyme, Nanjing, China). Transcriptome sequencing was conducted on the Illumina HiSeq Xten 2500 platform (Illumina, San Diego, CA, USA) with PE150. Raw data were filtered by FastQC and processed using bioinformatics analysis. Differentially expressed genes (DEGs) were identified using the DESeq2 algorithm with a $|Log2FC| > 1$ and $P < 0.05$ threshold. GO and KEGG pathway analyses were performed using the DAVID 6.8 bioinformatics database.

qRT-PCR assay

Total RNA was extracted from colonic tissues and Caco-2 cells using TRIzol reagent (Invitrogen). RNA was reverse transcribed to cDNA using a commercial cDNA Synthesis Kit (K1622, Thermo Fisher Scientific) on the PCR Amplifier (Life ECO-96, Bioer Technology, China). Quantitative real-time PCR was performed on an ABI-Q6 Real-Time PCR system using the 2× Master Mix kit (Roche, Switzerland). *GAPDH* was used as an internal reference, and the results were analyzed using the $2^{-\Delta\Delta CT}$ method. The primer sequences are provided in Supplemental Table 1. For qRT-PCR analyses, three independent experiments were performed using three biological replicates.

Cell counting kit- 8 (CCK8) assay

Caco-2 cell proliferation was evaluated using CCK8 (Beyotime) according to the manufacturer's instructions. Independent experiments were performed in triplicate. In brief, 10 μL of CCK8 reagent was added to each well after culturing for 24 h; the OD value was measured at 450 nm, and the following equation was used to quantify cell viability:

Cell viability (%) = [OD $_{value}$ treatment group × OD $_{value}$ no cell group]/[OD $_{value}$ control group ×OD $_{value}$ no cell group] ×100%.

Western blot analysis

Total protein was extracted from Caco-2 cells and lysed in an SDS buffer containing a proteinase inhibitor. The protein concentration was quantified using a bicinchoninic acid kit. Equal amounts of protein (20 μg) were separated using 10% SDS-PAGE and transferred onto a polyvinylidene difluoride membrane. The membranes were blocked with 5% skim milk at 25°C for 1 h. Next, primary antibodies against NNMT (1:1000, 15123-1-AP, Proteintech), NLRP3 (1:1000, FNab10120, Fintest, Wuhan, China), caspase-1 (1:500, Sc56036, Santa Cruz), GSDMD (1:1,000, A20728, ABclonal), RBP4 (1:1,000, 11774-1-AP, Proteintech), and GAPDH (1:2000, 60004-1-Lg, Proteintech) were incubated overnight at 4 °C. Subsequently, membranes were incubated with HRP-conjugated anti-rabbit IgG (1:20000, ab6721, Abcam, Boston, USA) and HRP-conjugated monoclonal mouse (1:1000, ab205719; Abcam) secondary antibodies at room temperature for 1 h. The obtained protein bands were captured using an enhanced chemiluminescence (ECL) system (Amersham Bioscience, Buckinghamshire, United Kingdom) equipped with an autoradiography film system and Image Kwile Quant (Dictionary Biosciences, LLC, USA). Independent experiments were performed in triplicate.

Enzyme-linked immunosorbent (ELISA) assay

Caco-2 cells were centrifuged at 1000 × *g* for 10 min, and the cell supernatant was extracted for analysis. Independent experiments were performed in triplicate. IL-1β and IL-18 levels were measured using an IL-1β ELISA kit (SEKH-0002, Solarbio, Beijing, China) and an IL-18 ELISA kit (ml058055, mlBio Technology), respectively,

according to the manufacturer's instructions. The OD value was measured at a wavelength of 450 nm.

Lactate dehydrogenase (LDH) activity assay

The LDH activity in the supernatants of Caco-2 cells was detected to assess cell injury using the LDH assay kit (BC0680, Solarbio) according to the manufacturer's instructions. Independent experiments were performed in triplicate.

Cell transfection

To assess the effects of NNMT and RBP4, Caco-2 cells were transfected with siRNA targeted to *NNMT* and *RBP4*, respectively. All siRNAs were synthesized by GenePharma (Supplementary Table 1). To overexpress NNMT, the full length NNMT sequence was cloned into pcDNA 3·1 vectors. Caco-2 cells were seeded into a six-well plate at a density of 3×10^5 cells/well and grown for 24 h in complete medium. The cells were rinsed with fresh media when the confluence rate reached 80–90% and then incubated with siRNA or vectors and Lipofectamine 2000 (Thermo Fisher Scientific) for 24 h to achieve transient transfection.

Methylated RNA immunoprecipitation (MeRIP) sequencing and MeRIP-qPCR

Total RNA was isolated from Caco-2 cells following NNMT overexpression and subjected to MeRIP. Briefly, the mRNA was fragmented and immunoprecipitated with anti-m6A magnetic beads from the riboMeRIPTMm6A Transcriptome profiling kit (C11051-1, RiboBio, China). For MeRIP sequencing, m6A-enriched RNA was used to prepare a cDNA library; the input RNA was also generated as a control. The samples were sequenced using the Nova-seq 6000 system (Illumina). Library preparation and high-throughput sequencing were performed by MivectorBio

(Shanghai, China). For MeRIP-qPCR, m6A-enriched RNA was used for qRT-PCR. For qRT-PCR analyses, three independent experiments were performed using three biological replicates.

Fluorescence in situ hybridization (FISH)

The IF/FISH double staining was adopted to detect the co-localization of RBP4 and NNMT. Paraffin sections of 21 CD patients were deparaffinized and rehydrated. The sections were soaked at room temperature in 3% H_2O_2 for 10 min to inactivate endogenous peroxidase. Freshly diluted gastric proteinase was dropped on the slices at room temperature for 10 min. Pre-hybridization solution and RBP4-specific probe were dropped on the slices and hybridized at 40°C overnight. The primary antibody NNMT (1:250, 15123-1-AP, Proteintech) and the secondary antibody Cy3-labeled goat anti-rabbit IgG(H+L) (A0516, Beyotime) were successively added for incubation. Nuclei were counterstained with DAPI. The slices were sealed with an anti-fade mounting medium for fluorescence. The results were observed under a fluorescence microscope and photographed.

Supplementary Tables

Supplementary Table 1. Primers used in this study

Gene	Primers (5' – 3')
GAPDH-F	AGAAGGCTGGGGCTCATT
GAPDH-R	AGAAGGCTGGGGCTCATT
DCLK1-F	GCTGATTTGACCCGAACTCTG
DCLK1-R	AGCCACATACATAACTCTCTCCT
B3GNT3-F	CAGCACGTTCAGAACTTCCTC
B3GNT3-R	GCGCACATAGTTGCTAGGGG
NNMT-F	TCTAAGCCATTTTAACCCTCGG
NNMT-R	TGTCAATCAGCAGGTCTCCC
HDAC9-F	AGTAGAGAGGCATCGCAGAGA
HDAC9-R	GGAGTGTCTTTCGTTGCTGAT
FUT3-F	CTGTCCCGCTGTTCAGAGATG
FUT3-R	AGGCGTGACTTAGGGTTGGA
DUSP4-F	GGCGGCTATGAGAGGTTTTCC
DUSP4-R	TGGTCGTGTAGTGGGGTCC
MGAT3-F	TAACCTGGTGTCCAGCTTTTTC
MGAT3-R	GAGTGGGAGTAGAGTGGGGTA
RBP4-F	CGACACAGACTACGACAC
RBP4-R	CCGCTGCCTTACAATCTT
EGR1-F	GGTCAGTGGCCTAGTGAGC
EGR1-R	GTGCCGCTGAGTAAATGGGA
CYP1A1-F	CACTCTTCCTTCGTCCCCTT
CYP1A1-R	TGGTTGATCTGCCACTGGTT
ST6GAL1-F	GTTACCACAGAGAAGCGCTT
ST6GAL1-R	TGGGGTGCAGCTTACGATAA
si-NNMT-1	CUAUGUGUGUGAUCUUGAATT<br>UUCAAGAUCACACACAUAGTT
si-NNMT-2	GCCUGUGACCUCAAUUAAATT<br>UUUAAUUGAGGUCACAGGCTT
si-NNMT-3	GCUCCUCUCUGCUUGUGAATT<br>UUCACAAGCAGAGAGGAGCTT
si-NC	UUCUCCGAACGUGUCACGUTT<br>ACGUGACACGUUCGGAGAATT
si-RBP4-1	CCAAGUUCAAGAUGAAGUATT

	UACUUCAUCUUGAACUUGGTT
si-RBP4-2	GCCUCUUUCUGCAGGACAATT<br>UUGUCCUGCAGAAAGAGGCTT
si-RBP4-3	GCUUCCGAGUCAAGGAGAATT<br>UUCUCCUUGACUCGGAAGCTT
si-NC	UUCUCCGAACGUGUCACGUTT<br>ACGUGACACGUUCGGAGAATT

Supplementary Table 2. General information of the included patients

**Clinical characteristics**	**Patients with CD (n = 102)**
**Gender**	
Male	85 (83.3%)
Female	17 (16.7%)
**Age**	27.52 ± 8.48
**Disease behavior**	
Non-stricturing and non-penetrating	69 (67.7%)
Stricturing	18 (17.6%)
Penetrating	15 (14.7%)
**CDAI score**	
> 150	19 (18.6%)
≤ 150	83 (81.4%)
**Disease location**	
Ileal	5 (4.9%)
Colonic	5 (4.9%)
Ileocolonic	78 (76.5%)
Upper gastrointestinal tract	14 (13.7%)
**History of intestinal surgery**	
Yes	16 (15.7%)
No	86 (84.3%)
**Perianal disease**	
Yes	63 (61.8%)
No	39 (38.2%)

Abbreviations: CD, Crohn's disease; CDAI: The Crohn's disease activity index.

Supplementary Figures

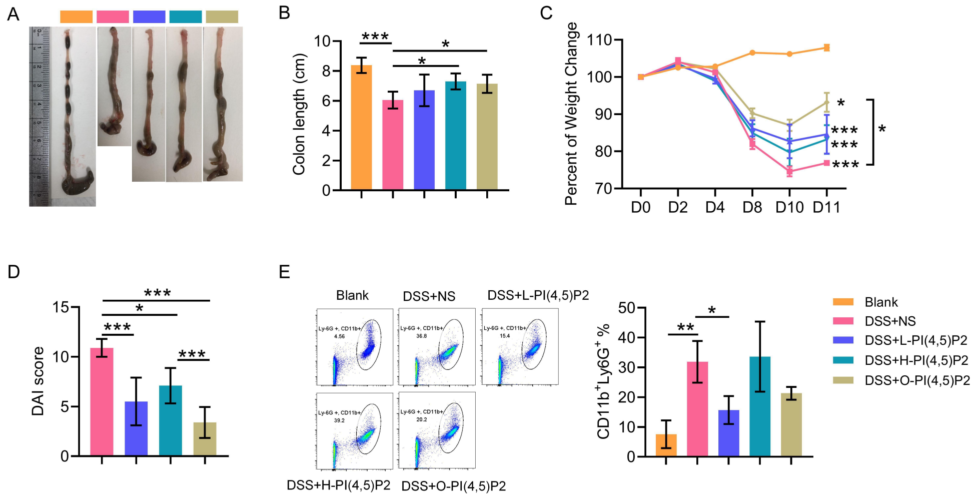

Supplementary Figure 1. The optimal PI(4,5)P2 treatment was 100 μmol/L PI(4,5)P2 enema. (A) Representative image of colon in each group. (B) The colon length of mice in each group. (C) The change of body weight of mice in each group. (D) DAI scores of colon in each group. (E) The distribution of neutrophils (Ly6G^{+}CD11b^{+}) in peripheral blood was determined by flow cytometry.

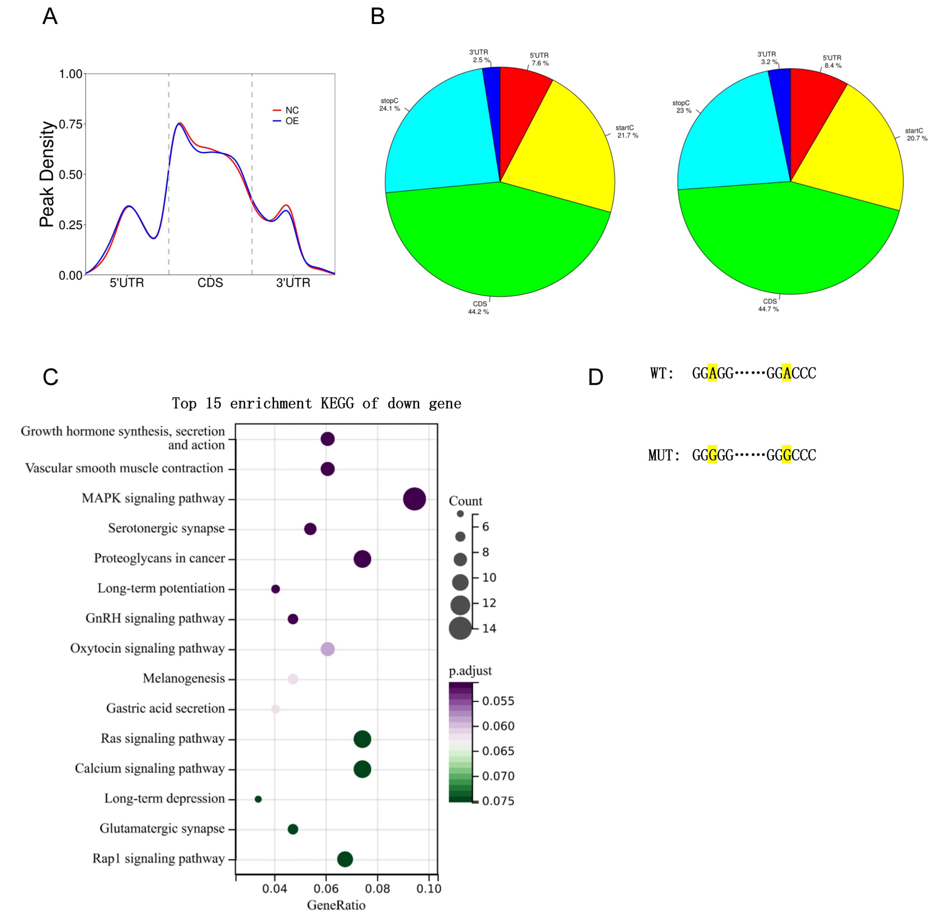

Supplementary Figure 2. MeRIP sequencing revealed the distribution of m6A peaks along with transcripts in Caco-2 cells after NNMT overexpression. (A) The distribution of m6A peaks density of transcripts in Caco-2 cells after transfected with blank vector or pcDNA3.1-NNMT. (B) Pie charts of distribution of m6A peaks in the whole transcriptome of Caco-2 cells from two groups. (C) The bubble plot of the top 15 of KEGG pathway enrichment. (D) Schematic representation of the m6A binding site mutation (A mutated to G) on RBP4.

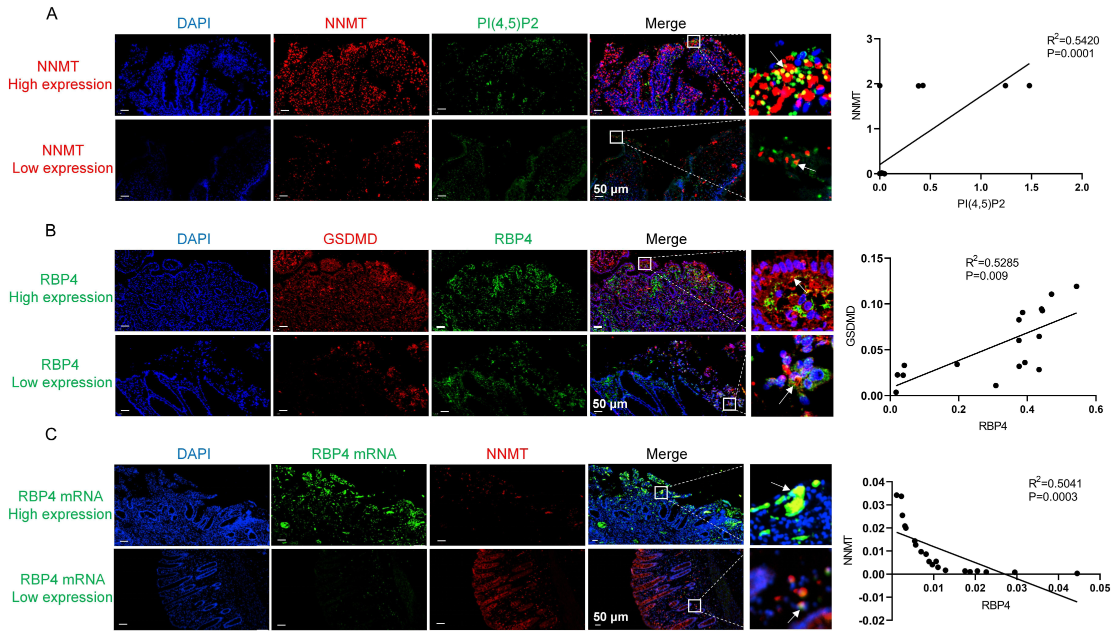

Supplementary Figure 3. Correlation analysis of PI(4,5)P2, NNMT, RBP4 and GSDMD in intestinal specimens of patients with CD. The expression correlation of PI(4,5)P2 with NNMT protein (A) and RBP4 protein with GSDMD protein (B) in the mucosa with inflammation lesions in patients with CD was assessed using IF. (C) The expression and co-localization of NNMT and RBP4 mRNA were detected by IF/FISH double staining. The white arrows represent co-localization. Scale bar, 50 μm; n = 21.
