# Supplementary figures and images for: PI(4,5)P2 alleviates colitis by inhibiting intestinal epithelial cell pyroptosis through NNMT-mediated RBP4 m6A modification
Source: Cell Death Dis. 2024 Dec 20;15(12):923. doi: 10.1038/s41419-024-07276-3 (PMC11661994; doi:10.1038/s41419-024-07276-3)

Figure 3G

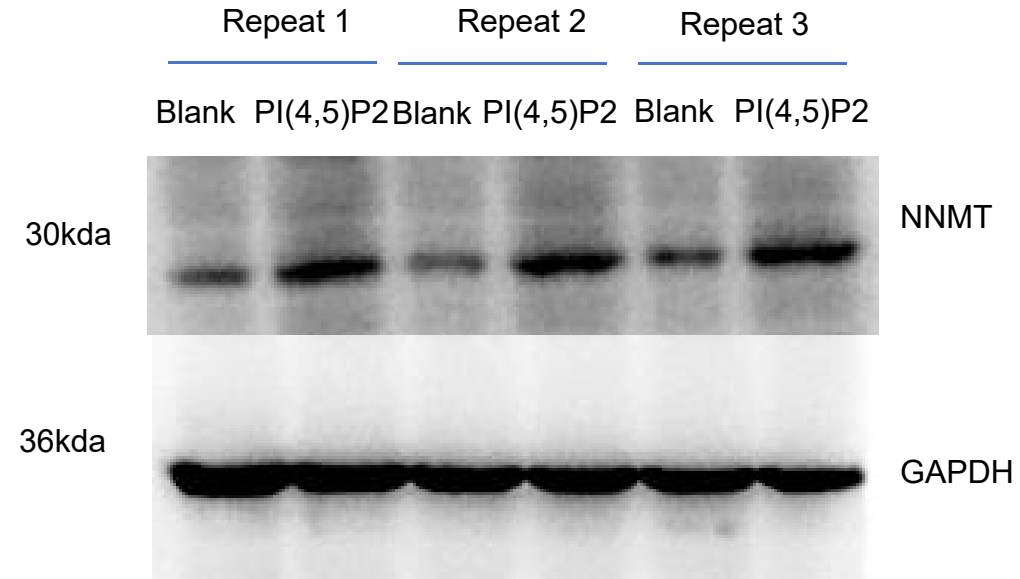

Figure 4B

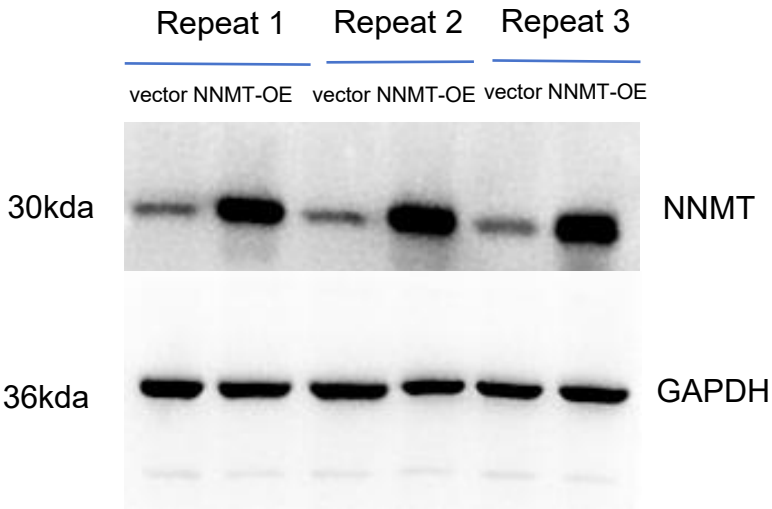

Figure 4G

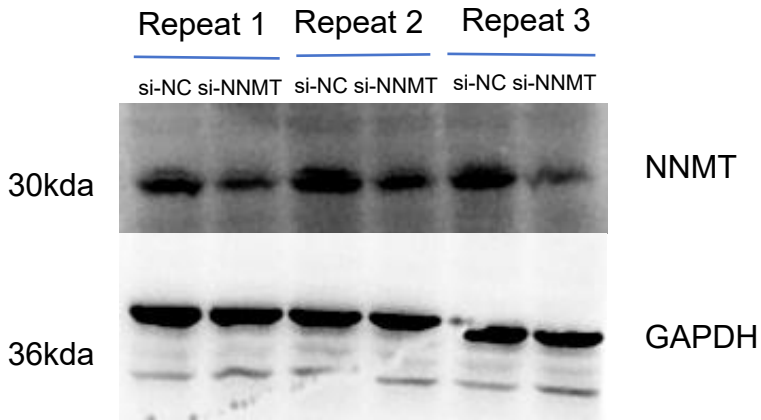

Figure 5H

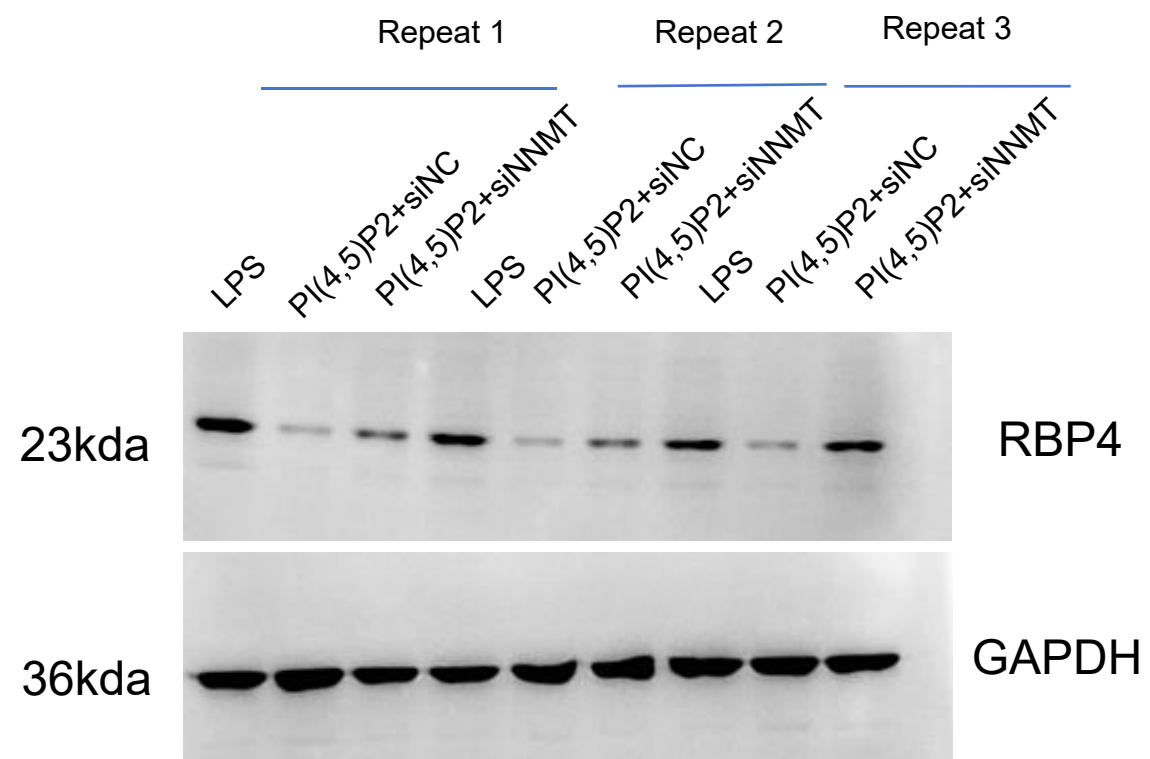

Figure 6C

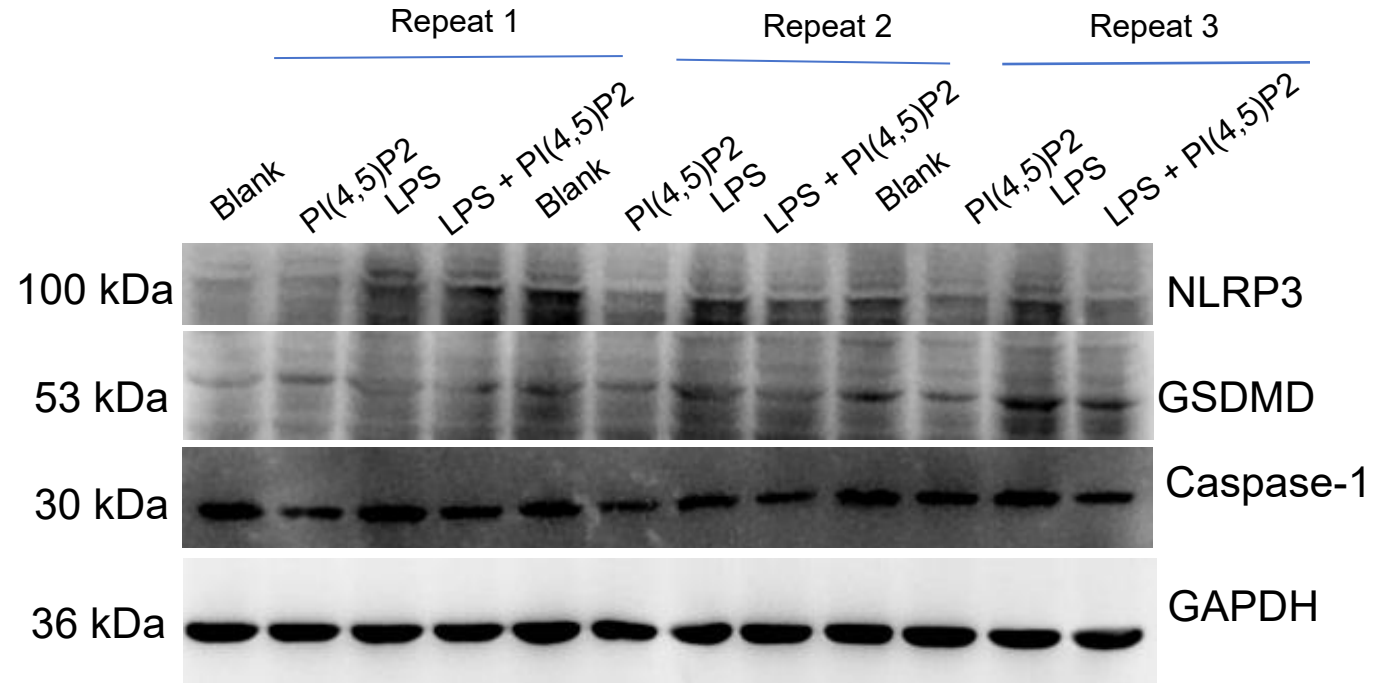

Figure 6F

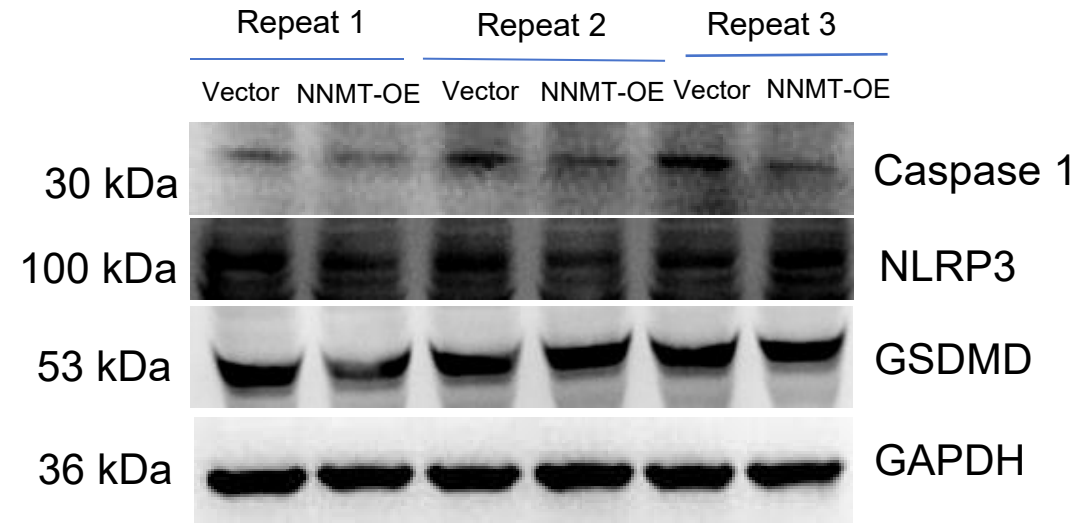

Figure 7A

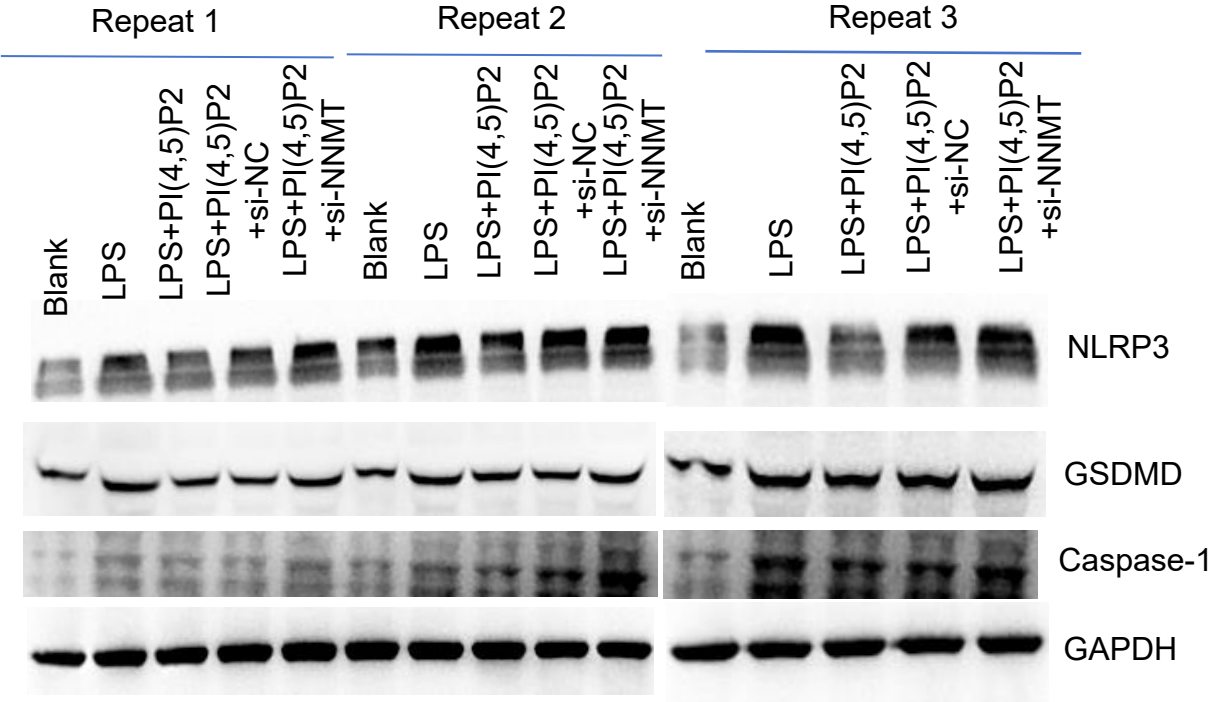

Figure 8B

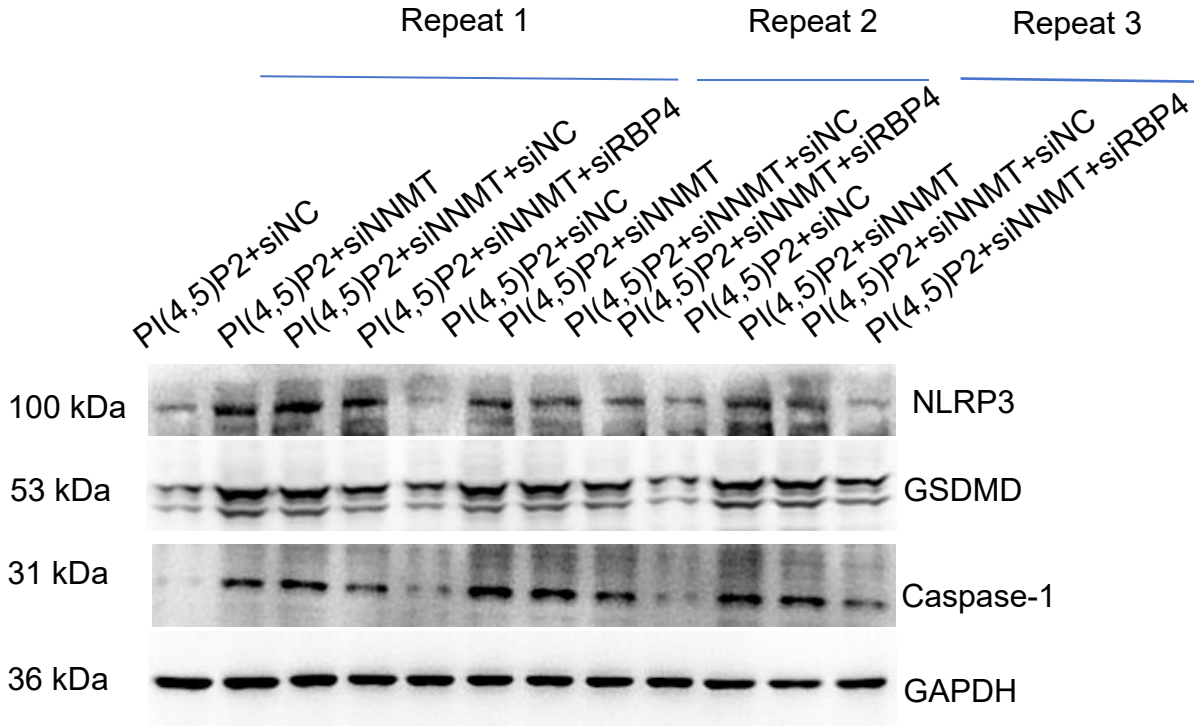

Supplement: Supplementary file 2 — Original Western blot images [file 41419_2024_7276_MOESM2_ESM.pdf]
